# Variability in Brain Structure and Function Reflects Lack of Peer Support

**DOI:** 10.1093/cercor/bhab109

**Published:** 2021-05-13

**Authors:** Matthias Schurz, Lucina Q Uddin, Philipp Kanske, Claus Lamm, Jérôme Sallet, Boris C Bernhardt, Rogier B Mars, Danilo Bzdok

**Affiliations:** 1 Donders Institute for Brain, Cognition and Behaviour, Radboud University Nijmegen, 6525 EN Nijmegen, The Netherlands; 2 Wellcome Centre for Integrative Neuroimaging, Department of Experimental Psychology, University of Oxford, Oxford OX1 3SR, UK; 3 Institute of Psychology, University of Innsbruck, 6020 Innsbruck, Austria; 4 Department of Psychology, University of Miami, Coral Gables, Florida 33124, USA; 5 Neuroscience Program, University of Miami Miller School of Medicine, Miami, Florida 33136, USA; 6 Clinical Psychology and Behavioral Neuroscience, Faculty of Psychology, Technische Universität Dresden, 01187 Dresden, Germany; 7 Max Planck Institute for Human Cognitive and Brain Sciences, 04103 Leipzig, Germany; 8 Social, Cognitive and Affective Neuroscience Unit, Department of Cognition, Emotion, and Methods in Psychology, Faculty of Psychology, University of Vienna, 1010 Vienna, Austria; 9 University of Lyon, Univ Lyon 1, INSERM, Stem Cell and Brain Research Institute U1208, 69500 Bron, France; 10 McConnell Brain Imaging Centre (BIC), Montreal Neurological Institute (MNI), McGill University, Montreal, Quebec H3A 2B4, Canada; 11 Wellcome Centre for Integrative Neuroimaging, Centre for Functional MRI of the Brain (FMRIB), Nuffield Department of Clinical Neurosciences, John Radcliffe Hospital, University of Oxford, Oxford OX3 9DU, UK; 12 Department of Biomedical Engineering, Faculty of Medicine, School of Computer Science, McGill University, Montreal, Quebec H3A 2B4, Canada; 13 Mila-Quebec Artificial Intelligence Institute, Montreal, Quebec H2S 3H1, Canada

**Keywords:** Bayesian hierarchical modeling, machine learning, population neuroscience, salience network, social brain

## Abstract

Humans are a highly social species. Complex interactions for mutual support range from helping neighbors to building social welfare institutions. During times of distress or crisis, sharing life experiences within one’s social circle is critical for well-being. By translating pattern-learning algorithms to the UK Biobank imaging-genetics cohort (*n* = ~40 000 participants), we have delineated manifestations of regular social support in multimodal whole-brain measurements. In structural brain variation, we identified characteristic volumetric signatures in the salience and limbic networks for high- versus low-social support individuals. In patterns derived from functional coupling, we also located interindividual differences in social support in action–perception circuits related to binding sensory cues and initiating behavioral responses. In line with our demographic profiling analysis, the uncovered neural substrates have potential implications for loneliness, substance misuse, and resilience to stress.

## Introduction

Compared with other species, human relationships are unique in their complexity. The quality and quantity of our daily encounters are critical for physical and mental health. Tight integration in groups and communities benefits our resilience during times of distress. Social embeddedness helps the immune system (Pressman et al. 2005), improves sleep quality (Kurina et al. 2011), and accelerates body tissue repair after injury (Reblin and Uchino 2008; Kim et al. 2016). In contrast, individuals who perceive themselves as socially disconnected are more prone to cognitive performance decline (Boss et al. 2015), Alzheimer’s-related dementias (Kuiper et al. 2015), and earlier death on average (Holt-Lunstad and Smith 2010; Steptoe et al. 2013). Indeed, in a meta-analysis of 148 epidemiological studies pooling across ~300 000 individuals, social embeddedness predicted mortality due to cardiovascular disease; and better so than factors like obesity, diet, alcohol consumption, or exercise (Holt-Lunstad and Smith 2010).

In the evolutionary lineage of primates and other species, accumulating research suggests a close link between the richness of one’s social environment and neocortex volume (Dunbar 1993, 1998). The sophistication of neurobiology may have coevolved with solving challenges posed by life in large social groups. In adult monkeys, experimentally increasing or decreasing the group size for daily peer interaction caused plasticity changes in features of brain anatomy (Sallet et al. 2011). Similarly, in experiments in humans, neuroplastic adaptations in social brain circuits were caused by regularly drawing on one’s social capacities through targeted training (Valk et al. 2017). Hence, both across-species and within-species evidence speaks to the flexible changes of functional brain architecture as a function of regular social exchange.

In our current time and age, humans live in social environments that are dramatically different from those of our primate ancestors. In our globalized and fast-paced world, different layers of regular social interaction can provide valuable input and crucial support. One such source is more spontaneous or loose interaction with acquaintances from our outer social circles (Granovetter 1973, 1983). However, the relationships in our inner social circle are most relevant for our psychological and physical well-being. People typically invest a substantial amount of their social efforts in only a handful of people—representing a person’s “support network” (Dunbar 2018). When asked whom someone would turn to in times of need of emotional, social, and economic aid, this group of close friends and family gets repeatedly mentioned (Sutcliffe et al. 2012). In married couples, the death of one spouse escalates the risk of death for the remaining partner. In ~400 000 married couples, mortality rates increased by 18% for widowed men, and by 16% for women who lost their spouse (Elwert and Christakis 2008). Further, the chance of becoming happy, depressed, or obese is directly mirrored by similar changes in our immediate peers. These mimetic effects were shown in a 20-year prospective study (Fowler and Christakis 2008). Even the amount of prosocial behavior can be predicted from the level of emotional resonance between people (Toi and Batson 1982). This constellation of findings highlights the importance of strong support ties with close friends and family (Dunbar 2018; Bzdok and Dunbar 2020).

Most recently, the COVID-19 pandemic has imposed an unprecedented disruption on the social support (SS) networks of many people. While unemployment rates have been rising, many countries have imposed restrictive measures for social distancing, or physical distancing. These public-health decisions have caused severe incisions on fluid social interaction in our everyday lives. The consequences are likely exacerbated for individuals who live in single-person households. Solitary living makes up >50% of the population in a growing number of metropolitan cities worldwide. This trend keeps increasing at a rapid pace (World Health Organization). Moreover, a survey reported that ~7% of Europeans were socially isolated already 15 years ago (Lelkes 2010): almost 1 in 10 Europeans admitted either never meeting friends or never meeting family outside of their own household. Not even once in the course of an entire year.

Importantly, in moments of sudden need, support ties cannot be just created “from scratch.” Building supportive relationships takes dedication, regular in-face encounters, and time investment over extended periods (Hall 2019). Such special relationships provide essential SS. Therefore, these interactions play a key role in buffering against distress and worries in times of crisis or uncertainty (Cohen and Syme 1985; Zaki and Williams 2013). For example, people with adequate SS show lower daily levels of the stress hormone cortisol than people with less backing by friends and family (Evolahti et al. 2006).

Special friends and family play a central support role. There is hence a knowledge gap regarding the brain substrates of regular SS. While the neural implications of living in large social groups have been repeatedly characterized, little is known regarding the effect of quality and closeness of these relationships. According to behavioral research, different facets of human relations are associated with different types of social information processing (Kardos et al. 2017; Morelli et al. 2017). In the human brain, social information is processed by several distinct neural systems. Some of the responsive neural systems are not exclusively linked to social cognition (Spunt and Adolphs 2017; Alcalá-López et al. 2018). Based on a quantitative meta-analysis of 188 brain imaging studies featuring 4207 participants (Schurz et al. 2021), we recently showed that many different kinds of social processes mainly recruit two large-scale brain networks: One neural system largely corresponds to the so-called default mode network (DMN). This set of brain regions is believed to subserve more cognitive and reasoning-based forms of social cognition, such as taking others’ perspectives (Frith and Frith 2006; Adolphs 2009). The other neural system, including the so-called salience network, is implicated in more affective processes based on action–perception circuits. This cohesive set of brain regions is involved in emotionally connecting to others, including empathic capacities for affective sharing (Preston and de Waal 2002; de Vignemont and Singer 2006).

Much evidence on these two brain systems for social processing is based on neural activity responses to static visual screen cues in strictly controlled experimental settings. Since recently, researchers are increasingly translating more naturalistic and “real-life” forms of social cognition into brain imaging experiments. By putting a premium on ecological validity, imaging neuroscience studies increasingly unmasked more complex and rich patterns of brain activity (Redcay and Schilbach 2019).

For these reasons, the present study aimed to clarify which neural systems are linked to day-to-day engagements within SS circles. We capitalized on the UK Biobank imaging-genetics population cohort because it offers rich phenotypical details (*n* = ~40 000), including multimodal brain imaging data and participants’ indicators of real-life social interactions. Specifically, we used the frequency of exchange of confidential information as a proxy for the amount of emotionally significant social contact in everyday life.

## Materials and Methods

### Data Resources

The UK Biobank is a prospective epidemiology resource that offers extensive behavioral and demographic assessments, medical and cognitive measures, as well as biological samples in a cohort of ~500 000 participants recruited from across Great Britain (https://www.ukbiobank.ac.uk/). This openly accessible population dataset aims to provide multimodal brain-imaging for ~100 000 individuals, planned for completion in 2022. The present study was based on the recent data release from February 2020 that augmented brain scanning information to ~40 000 participants.

In an attempt to improve comparability and reproducibility, our study built on the uniform data preprocessing pipelines designed and carried out by FMRIB, Oxford University, UK (Alfaro-Almagro et al. 2018). Our study involved data from the ~40 000 participant release with brain-imaging measures of gray matter morphology (T1-weighted MRI [sMRI]) and neural activity fluctuations (resting-state functional MRI [fMRI]) from 47.5% men and 52.5% women, aged 40–69 years when recruited (mean age 54.9, standard deviation [SD] 7.5 years). Our study focused on trait SS as a measure of the frequency of social interactions (Hawkley et al. 2003; Luhmann and Hawkley 2016; Bzdok and Dunbar 2020). This self-reported item was based on the following question: “How often are you able to confide in someone close to you?”. Our study distinguished between people reporting engagement in “daily or almost daily” SS (treated as positive case, 1) or confiding in others less often (treated as negative case, 0).

Corresponding measures are found in widely used assessments of social embeddedness (Cohen and Hoberman 1983; Hawkley et al. 2005; Cyranowski et al. 2013). For example, the Social Relationships Scales of the NIH Toolbox (Cyranowski et al. 2013) feature the dimension of emotional SS, which closely resembles our measure of SS. This dimension holds items, such as “I have someone I trust to talk with about my problems”, or “I can get helpful advice from others when dealing with a problem”. Conceptually similar dimensions (Cyranowski et al. 2013) are also featured in other standard measurement-tools of social embeddedness, such as the Revised UCLA Loneliness Scale (Hawkley et al. 2005) and the Interpersonal Support Evaluation List (Cohen and Hoberman 1983).

A variety of studies found single-item measures of social traits to be reliable and valid (e.g., Mashek et al. 2007; Dollinger and Malmquist 2009; Atroszko et al. 2015). For example, the single item “There are people I can talk to” correlates highly (*r* = 0.88) with the R-UCLA Loneliness Scale dimension that resembles SS (Hawkley et al. 2005). Previous research successfully used individual items for measuring SS (Atroszko et al. 2015), community connectedness (Mashek et al. 2007), and perceived social isolation (Ong et al. 2016).

Studying SS as low versus high is following strategies used in previous behavioral studies (Le et al. 2016; Sasaki et al. 2019), and comparable with previous analyses of UK Biobank data in genetic (Day et al. 2018) and epidemiological (Elovainio et al. 2017) studies. The latter epidemiological UK Biobank study (Elovainio et al. 2017) provides additional details on demographic characteristics of UK Biobank participants with high versus low SS. The present analyses were conducted under UK Biobank application number 25163. All participants provided informed consent. Further information on the consent procedure can be found elsewhere (http://biobank.ctsu.ox.ac.uk/crystal/field.cgi?id=200).

### Multimodal Brain-Imaging and Preprocessing Procedures

Magnetic resonance imaging (MRI) scanners (3 T Siemens Skyra) were matched at several dedicated imaging sites with the same acquisition protocols and standard Siemens 32-channel radiofrequency receiver head coils. To protect the anonymity of the study participants, brain-imaging data were defaced and any sensitive metainformation was removed. Automated processing and quality control pipelines were deployed (Miller et al. 2016; Alfaro-Almagro et al. 2018). To improve homogeneity of the imaging data, noise was removed by means of 190 sensitivity features. This approach allowed for the reliable identification and exclusion of problematic brain scans, such as due to excessive head motion.

#### Structural MRI

The sMRI data were acquired as high-resolution T1-weighted images of brain anatomy using a 3D MPRAGE sequence at 1 mm isotropic resolution. Preprocessing included gradient distortion correction (GDC), field of view reduction using the Brain Extraction Tool (Smith 2002) and FLIRT (Jenkinson and Smith 2001; Jenkinson et al. 2002), as well as nonlinear registration to MNI152 standard space at 1 mm resolution using FNIRT (Andersson et al. 2007). To avoid unnecessary interpolation, all image transformations were estimated, combined, and applied by a single interpolation step. Tissue-type segmentation into cerebrospinal fluid (CSF), gray matter (GM), and white matter (WM) was applied using FAST (FMRIB’s Automated Segmentation Tool, (Zhang et al. 2001)) to generate full bias-field-corrected images. SIENAX (Smith et al. 2002), in turn, was used to derive volumetric measures normalized for head sizes.

#### Functional MRI

**Figure 1:**
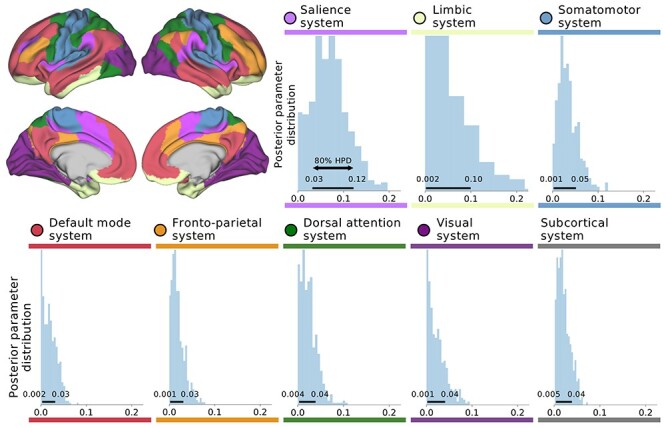
Gray matter variation across specific large-scale brain systems explains strong effects related to social support (SS). At the network level, our Bayesian hierarchical modeling framework directly estimated the varying effects of entire brain networks in explaining high versus low SS in the UK Biobank participants. The fully probabilistic modeling approach allowed volume variation effects to be estimated jointly in separate brain regions (see Fig. 2) and spatially distributed networks of constituent brain regions (shown in this figure). In rough analogy to ANOVA, the network definitions could be viewed as factors and the region definitions could be viewed as continuous factor levels. This analysis tactic enabled quantifying the extent to which spatially dispersed regional variation in gray matter volume can be coherently explained by differences among major brain networks (Bzdok et al. 2020; Kiesow et al. 2020). Histograms show marginal posterior distributions of the overall explanatory variance (sigma parameter) for each brain network (volume measures in standard units). Horizontal black bars indicate the highest-posterior density (HPD) interval of the model’s network variance parameters, ranging from 10 to 90% probability. Population-level volume variation in the salience and limbic networks emerged as preferentially linked to interindividual differences in SS. Note that the subcortical network is not shown in the cortical surface view.

**Figure 2:**
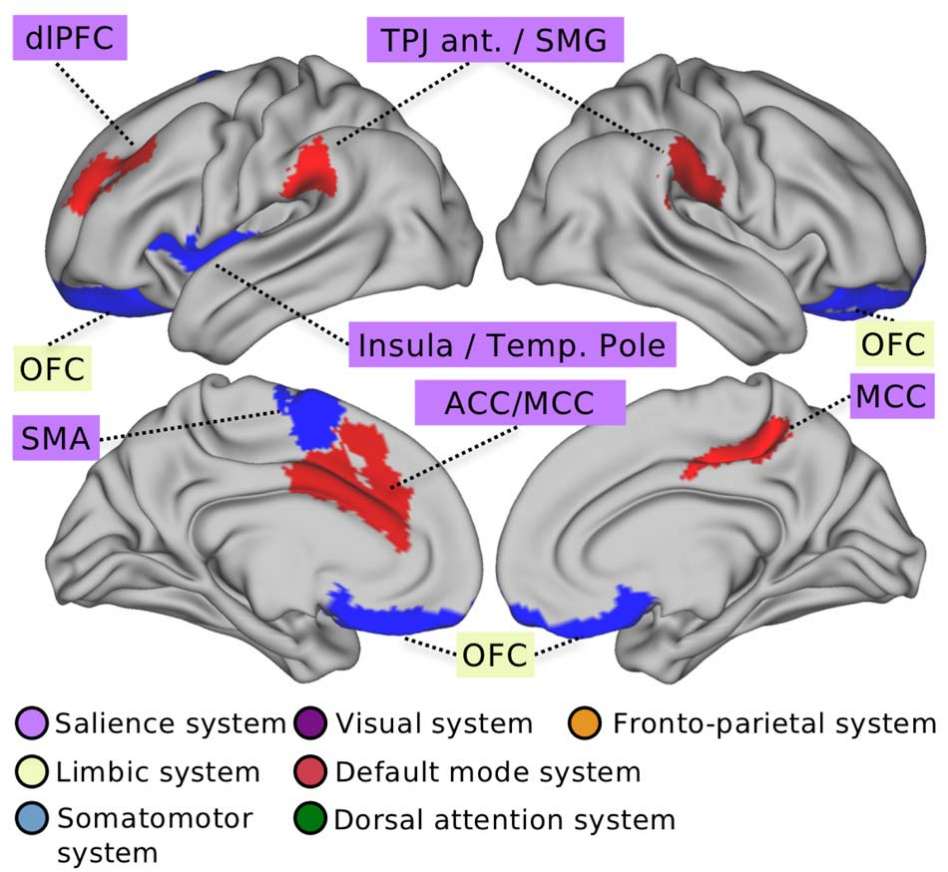
Top brain regions that explain gray matter differences related to SS. At the region level, our Bayesian hierarchical modeling framework identified for which brain regions variability in gray matter volume explains the level of SS reported by the participants. Strongest associations to day-to-day SS (cf. Fig. 1) were determined based on effect sizes (mean parameters) of the marginal posterior parameter distributions (volume measures in standard units). Key region associations were located in parts of the salience network, including the anterior insula and anterior/mid cingulate cortex. Additional neural substrates of SS were located in regions of the limbic network, including the OFC. Red (blue) color indicates positive (negative volume) effects related to regular SS, for the top 10 regions found in this analysis. Abbreviations: ACC = anterior cingulate cortex; dlPFC = dorso-lateral prefrontal cortex; MCC = midcingulate cortex; OFC = orbitofrontal cortex; SMA = supplementary motor area; SMG = supramarginal gyrus; Temp. Pole = temporal pole; TPJ ant = anterior portion of the TPJ. The subcortical system is not shown in this view.

**Figure 3:**
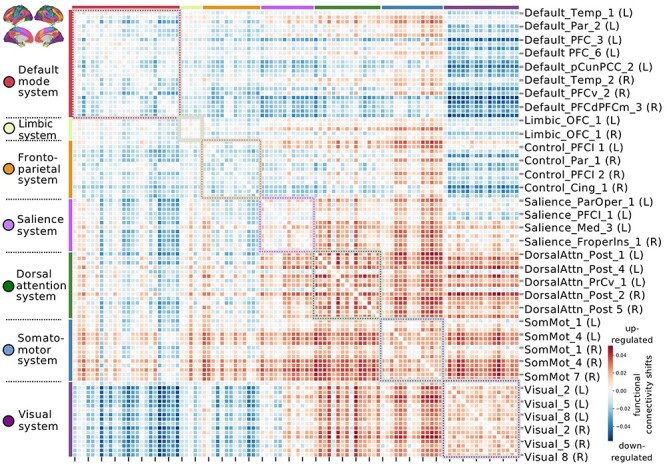
Leading functional coupling signature of SS suggests up-regulated action-perception systems and down-regulated interplay with internal-cognition systems. Functional connectivity shifts are shown for the dominant population mode related to everyday SS (connectivity relevancies in standard units). Statistical significance of this population mode of coherent functional coupling differences in high versus low SS participants was determined by nonparametric permutation testing (*P* < 0.05). Red connectivity links indicate compounded functional coupling for individuals with high amounts of regular SS, which suggests an up-regulation of “here-and-now” related networks, including the salience, dorsal attention, somatomotor, visual networks. Blue connectivity links indicate reduced coupling between regions in high SS.

The fMRI data of intrinsic neural activity were acquired without engagement in a predefined experimental task context at 2.4 mm spatial resolution, time to repeat = 0.735 s, and with multiband acceleration of 8. A single-band reference image with higher between-tissue contrast and without T1-saturation effects was acquired within the same geometry as the time series of neural activity maps. The reference scan was used for the alignment to other brain-imaging modalities and correction for head motion. Preprocessing was performed using MELODIC (Beckmann and Smith 2004), including EPI and GDC unwarping, motion correction, grand-mean intensity normalization, and high-pass temporal filtering (Gaussian-weighted least-squares straight line fitting, sigma = 50 s). The ensuing images were submitted to motion correction using MCFLIRT (Jenkinson et al. 2002). Structured artifacts were removed by combining ICA and FMRIB’s ICA-based X-noiseifier (Griffanti et al. 2014). To help reduce unnecessary interpolation effects, all intermediate warp operations were merged into a composite transformation allowing for simultaneous application to fMRI maps. For the display of results (see Figs 1–3), maps were projected to the cortical surface. This was done via volume-to-surface mapping in wb_command (www.humanconnectome.org), based on the Human Connectome Project (HCP) group average template “S1200_MSMAll.”

### Analysis of Associations between Social Support and Gray Matter Patterns

Neurobiologically interpretable measures of gray matter volume were extracted in all participants by summarizing whole-brain sMRI maps in Montreal Neurological Institute (MNI) reference space. This feature generation step was guided by the topographical brain region definitions of the widely used Schaefer-Yeo atlas comprising 100 parcels (Schaefer et al. 2018) as well as the 15 subcortical parcels of the Harvard-Oxford atlas (Desikan et al. 2006). The derived quantities of local gray matter morphology comprised 115 volume measures for each participant. The participant-level brain region volumes provided the input variables for our Bayesian hierarchical modeling approach (cf. below). As a data-cleaning step, interindividual variation in brain region volumes that could be explained by variables of no interest were regressed out: body mass index, head size, average head motion during task-related brain scans, average head motion during task-unrelated brain scans, head position and receiver coil in the scanner (*x*, *y*, and *z*), position of scanner table, as well as the data acquisition site.

To examine population variation of our atlas regions in the context of regular SS, a purpose-designed Bayesian hierarchical model was a natural choice of method, building on our previous research (Bzdok et al. 2017, 2020; Kiesow et al. 2020; Spreng et al. 2020). In contrast, classical linear regression combined with statistical significance testing would simply have provided *P*-values against the null hypothesis of no difference between high-SS and low-SS participants in each brain region. Instead of limiting our results and conclusions to strict categorical statements, each region being either relevant for differences in SS or not, our analytical strategy aimed at full probability distributions that expose how brain region volumes converge or diverge in their relation to SS as evidenced in the UK Biobank population. In a mathematically rigorous way, our approach estimated coherent, continuous estimates of uncertainty for each model parameter at play for its relevance in SS. Our study thus addressed the question “How certain are we that a regional brain volume is divergent between high and low social support individuals?”. Our analysis did not ask “Is there a strict categorical difference in region volume between high and low social support individuals?”

The elected Bayesian hierarchical framework also enabled simultaneous modeling of multiple organizational principles: (i) *segregation* into separate brain regions and (ii) *integration* of groups of brain regions in form of spatially distributed brain networks. Two regions of the same atlas network are more likely to exhibit similar volume effects than two regions belonging to two separate brain networks. Each of the region definitions was preassigned to one of the seven large-scale network definitions in the Schaefer-Yeo atlas (Schaefer et al. 2018) or the collection of subcortical regions from the Harvard-Oxford atlas (Desikan et al. 2006), providing a native multilevel structure. Setting up a hierarchical generative process enabled our analytical approach to borrow statistical strength between model parameters at the higher network level and model parameters at the lower level of constituent brain regions. By virtue of exploiting partial pooling, the brain region parameters were modeled themselves by the hyperparameters of the hierarchical regression as a function of the network hierarchy to explain SS. Assigning informative priors centered around zero provided an additional form of regularization by shrinking coefficients to zero in the absence of evidence to the contrary. We could thus provide fully probabilistic answers to questions about the morphological relevance of individual brain locations and distributed cortical networks by a joint varying-effects estimation that profited from several biologically meaningful sources of population variation.

The model specification placed emphasis on careful inference of unique posterior distributions of parameters at the brain network level to discriminate individuals with weak SS (encoded as outcome 0) and strong SS (outcome 1):

$$ y\sim \mathrm{Bernoulli}\left(\ p\right) $$

$$ \mathrm{logit}(p)={x}_1\ast{\beta}_{{\mathrm{region}}_1}+\dots +{x}_p\ast{\beta}_{{\mathrm{region}}_p}+{\alpha}_{\mathrm{men}\left[\mathrm{sex}\right]}+ $$

$$ {\alpha}_{\mathrm{women}\left[\mathrm{sex}\right]}+{\alpha}_{\mathrm{men}\_\mathrm{age}\left[\mathrm{sex}\right]}\ast{\mathrm{age}}_{\mathrm{men}}+{\alpha}_{\mathrm{women}\_\mathrm{age}\left[\mathrm{sex}\right]}\ast{\mathrm{age}}_{\mathrm{women}} $$

$$\begin{array}{l} {\beta}_{{\mathrm{region}}_{\mathrm{network}\_\mathrm{Visual}}}\sim \mathrm{MVNormal}\left(\left[\begin{array}{c}0\\{}\vdots \\{}0\end{array}\right],{\Sigma}_{\mathrm{Visual}}\right);\\{\Sigma}_{\mathrm{Visual}}=\left[\begin{array}{ccc}{\sigma}_o^2& \cdots & \\{}\vdots & \ddots & \vdots \\{}& \cdots & {\sigma}_o^2\end{array}\right] \end{array}$$

$$\begin{array}{l} {\beta}_{{\mathrm{region}}_{\mathrm{netwok}\_\mathrm{SomMot}}}\sim \mathrm{MVNormal}\left(\left[\begin{array}{c}0\\{}\vdots \\{}0\end{array}\right],{\Sigma}_{\mathrm{SomMot}}\right);\\{\Sigma}_{\mathrm{SomMot}}=\left[\begin{array}{ccc}{\sigma}_p^2& \cdots & \\{}\vdots & \ddots & \vdots \\{}& \cdots & {\sigma}_p^2\end{array}\right] \end{array}$$

$$\begin{array}{l} {\beta}_{{\mathrm{region}}_{\mathrm{network}\_\mathrm{Limbic}}}\sim \mathrm{MVNormal}\left(\left[\begin{array}{c}0\\{}\vdots \\{}0\end{array}\right],{\Sigma}_{\mathrm{Limbic}}\right);\\{\Sigma}_{\mathrm{Limbic}}=\left[\begin{array}{ccc}{\sigma}_q^2& \cdots & \\{}\vdots & \ddots & \vdots \\{}& \cdots & {\sigma}_q^2\end{array}\right] \end{array}$$

$$\begin{array}{l} {\beta}_{{\mathrm{region}}_{\mathrm{network}\_\mathrm{Salience}}}\sim \mathrm{MVNormal}\left(\left[\begin{array}{c}0\\{}\vdots \\{}0\end{array}\right],{\Sigma}_{\mathrm{Salience}}\ \right);\\{\Sigma}_{\mathrm{Salience}}=\left[\begin{array}{ccc}{\sigma}_r^2& \cdots & \\{}\vdots & \ddots & \vdots \\{}& \cdots & {\sigma}_r^2\end{array}\right] \end{array}$$

$$\begin{array}{l} {\beta}_{{\mathrm{region}}_{\mathrm{network}\_\mathrm{Control}}}\sim \mathrm{MVNormal}\left(\left[\begin{array}{c}0\\{}\vdots \\{}0\end{array}\right],{\Sigma}_{\mathrm{Control}}\ \right);\\{\Sigma}_{\mathrm{Control}}=\left[\begin{array}{ccc}{\sigma}_s^2& \cdots & \\{}\vdots & \ddots & \vdots \\{}& \cdots & {\sigma}_s^2\end{array}\right] \end{array}$$

$$\begin{array}{l} {\beta}_{{\mathrm{region}}_{\mathrm{network}\_\mathrm{DorsalAttn}}}\sim \mathrm{MVNormal}\left(\left[\begin{array}{c}0\\{}\vdots \\{}0\end{array}\right],{\Sigma}_{\mathrm{DorsalAttn}}\ \right);\\{\Sigma}_{\mathrm{DorsalAttn}}=\left[\begin{array}{ccc}{\sigma}_t^2& \cdots & \\{}\vdots & \ddots & \vdots \\{}& \cdots & {\sigma}_t^2\end{array}\right] \end{array}$$

$$\begin{array}{l} {\beta}_{{\mathrm{region}}_{\mathrm{network}\_\mathrm{Default}}}\sim \mathrm{MVNormal}\left(\left[\begin{array}{c}0\\{}\vdots \\{}0\end{array}\right],{\Sigma}_{\mathrm{Default}}\ \right);\\{\Sigma}_{\mathrm{Default}}=\left[\begin{array}{ccc}{\sigma}_u^2& \cdots & \\{}\vdots & \ddots & \vdots \\{}& \cdots & {\sigma}_u^2\end{array}\right] \end{array}$$

$$\begin{array}{l} {\beta}_{{\mathrm{region}}_{\mathrm{network}\_\mathrm{Subcortical}}}\sim \mathrm{MVNormal}\left(\left[\begin{array}{c}0\\{}\vdots \\{}0\end{array}\right],{\Sigma}_{\mathrm{Subcortical}}\ \right);\\{\Sigma}_{\mathrm{Subcortical}}=\left[\begin{array}{ccc}{\sigma}_v^2& \cdots & \\{}\vdots & \ddots & \vdots \\{}& \cdots & {\sigma}_v^2\end{array}\right] \end{array}$$

$$ {\alpha}_{\mathrm{men}}\sim \mathcal{N}\left(0,1\right) $$

$$ {\alpha}_{\mathrm{women}}\sim \mathcal{N}\left(0,1\right) $$

$$ {\alpha}_{\mathrm{men}\_\mathrm{age}}\sim \mathcal{N}\left(0,1\right) $$

$$ {\alpha}_{\mathrm{women}\_\mathrm{age}}\sim \mathcal{N}\left(0,1\right) $$

where sigma parameters estimated the overall variance across the *p* brain regions that belong to a given atlas network, independent of whether the volume effects of the respective constituent brain regions had positive or negative direction. As such, the network variance parameters sigma directly quantified the magnitude of intranetwork coefficients, and thus the overall relevance of a given network in explaining regular SS based on the dependent region morphology measures. All regions belonging to the same brain network shared the same variance parameter in the diagonal of the covariance matrix, while off-diagonal covariance relationships were zero.

Probabilistic posterior distributions for all model parameters were estimated for the hierarchical models. Our Bayesian approach could thus simultaneously appreciate gray matter variation in segregated brain regions as well as in integrative brain networks in a population cohort. The approximation of the posterior distributions was carried out by the NUTS sampler (Gelman et al. 2014), a type of Markov chain Monte Carlo (MCMC), using the PyMC3 software (Salvatier et al. 2016). After tuning the sampler for 4000 steps, we drew 1000 samples from the joint posterior distribution over the full set of parameters in the model for analysis. Proper convergence was assessed by ensuring Rhat measures (Gelman et al. 2014) stayed below 1.02.

### Analysis of Associations between Social Support and Functional Connectivity Patterns

Quantitative measures of functional connectivity were computed for cortex-wide brain regions as defined by the Schaefer-Yeo atlas (Schaefer et al. 2018). Functional connectivity profiles for each participant were derived by computing Pearson’s correlation between their neural activity fluctuations. To this end, in each participant, the time series of whole-brain fMRI signals, obtained in the absence of an externally structured experimental task, were summarized by averaging for each brain region in the atlas. The approach yielded the functional coupling signature of the whole cortex as a 100 x 100 region coupling matrix for each participant. The ensuing region–region coupling estimates underwent standardization across participants by centering to zero mean and unit scaling to a variance of one (cf. next step). Interindividual variation in the functional coupling strengths between brain regions that could be explained by variables of no interest were regressed out in a data-cleaning step: body mass index, head size, average head motion during task-related brain scans, average head motion during task-unrelated brain scans, head position as well as receiver coil in the scanner (*x*, *y*, and *z*), position of scanner table, and data acquisition site, as well as age, sex and age–sex interactions.

We then sought the dominant coupling regime—“mode” of population covariation—that provides insight into how functional variability in 100 brain regions can explain regular SS. Partial least squares (PLS) was an ideal analytical method to decompose the obtained 100 x 100 matrix of functional coupling fingerprints with respect to SS. The variable set *X* was constructed from the lower triangle of the participants’ functional coupling matrices. The target vector *y* encoded more socially engaged participants as +1 and less socially engaged participants as −1. PLS involves finding the matrix factorization into *k* low-rank brain representations that maximize the correspondence with our social trait of interest. PLS thus identified the matrix projection that yielded maximal covariance between sets of region couplings in the context of participant reports of SS.

In other words, the extracted functional coupling mode identified the driving linear combinations of cortical brain connections that featured the best correspondence to regular SS. Concretely, positive (negative) modulation weights revealed increased (decreased) correlation strengths, relative to average functional coupling. This is because the computed functional connectivity estimates were initially normalized to zero mean and unit variance across participants. For example, a functional connectivity input into PLS of 0 denoted the average functional coupling strength in our UK Biobank sample, rather than an absence of functional connectivity between the region pair. The derived pattern of PLS weights, or canonical vectors, thus indicated deviations from average functional coupling variation in our cohort. Moreover, the variable sets were entered into PLS after a confound-removal procedure (cf. above).

Next, we assessed the statistical robustness of the resulting dominant PLS mode of functional coupling deviations related to SS in a nonparametric permutation procedure, following previous research (Miller et al. 2016). Relying on minimal modeling assumptions, a valid empirical null distribution was derived for the Pearson’s correlation between low-rank projections of the dominant mode resulting from PLS analysis. In 1000 permutation iterations, the functional connectivity matrix was held constant, while the SS labels were submitted to random shuffling. The constructed surrogate datasets preserved the statistical structure idiosyncratic to the fMRI signals, yet permitted to selectively destroy the signal properties that are related to SS (Efron 2012). The generated distribution of the test statistic reflected the null hypothesis of random association between the brain’s functional coupling and amount of regular SS across participants. We recorded the Pearson’s correlations *r* between the perturbed low-rank projections in each iteration. *P*-value computation was based on the 1000 Pearson’s *r* estimates from the null PLS model.

### Demographic Profiling Analysis of the Brain Correlates of Social Support

We finally performed a profiling analysis of the brain regions that were most strongly associated with regular SS. Separately in brain structure and function, we carried out a rigorous test for multivariate associations between our top regions and a diverse set of indicators that exemplify the domains of (a) basic demographics, (b) personality features, (c) substance-use behaviors, and (d) social network properties (Table 1; for details, see https://www.ukbiobank.ac.uk/data-showcase/). The set of behavioral variables and the set of brain measures were *z*-scored across participants to conform to zero mean and unit variance. The brain variables were submitted to the top 10 (sMRI) or 10% (fMRI) of brain measures that were identified as most important in the context of SS (cf. above). In the case of brain structure, the target brain regions were selected based on the (absolute) modes of the Bayesian posteriors of marginal parameter distributions at the region level (cf. above). In the case of brain function, the target brain connections were selected based the (absolute) effect sizes from the dominant PLS mode (cf. above).

**Table 1:** Sociodemographic characteristics for individuals with high versus low SS

		UK Biobank ID 2110	
UK Biobank ID	Description	Regular social support	Lack of social support
	Overall participants	55%	45%
31	Sex (men:women)	47:53%	48:52%
21 022	Age (years)	54.73 ( ±7.45 SD)	55.03 (±7.49 SD)
845	Age completed school education (years)	17.07 (±2.74 SD)	17.06 (±2.84 SD)
34	Year of birth	1953.28 (±7.43 SD)	1953.03 (±7.47 SD)
728	Number of vehicles in household	2.80 (±0.80 SD)	2.63 (±0.84 SD)
738	Average total household income before tax (5 = high, 1 = low)	3.11 (±1.10 SD)	2.82 (±1.15 SD)
6142	Employment status (payed full time job = 1, not = 0)	70:30%	69:31%
20 016	Fluid intelligence score (0 = low, 13 = high)	6.66 (±2.00 SD)	6.73 (±2.07 SD)
4548	Health satisfaction (1 = extremely happy, 6 = extremely unhappy)	2.53 (±0.78 SD)	2.69 (±0.82 SD)
699	Length of time at current address (years)	16.63 (±10.42 SD)	16.87 (±11.00 SD)
1070	Time spent watching television (hours per day)	2.50 (±1.36 SD)	2.57 (±1.44 SD)
1080	Time spent using computer (hours per day)	1.46 (±1.46 SD)	1.54 (±1.56 SD)
1110	Length of mobile phone use (0 = never, 4 = more than 8 years)	2.82 (±1.30 SD)	2.77 (±1.34 SD)
1130	Hands-free device/speakerphone use with mobile phone in last 3 month (0 = never, 1 = always)	0.38 (±0.89 SD)	0.37 (±0.87 SD)
1628	Alcohol intake versus 10 years previously (1 = more now, 3 = less now)	0.38 (±0.89 SD)	0.37 (±0.87 SD)
20 403	Amount of alcohol drunk on a typical drinking day	2.20 (±0.74 SD)	2.23 (±0.74 SD)
1558	Alcohol intake frequency (1 = almost daily, 6 = never)	2.63 (±1.37 SD)	2.75 (±1.42 SD)
826	Job involves shift work (1 = never, 4 = always)	1.26 (±0.76 SD)	1.30 (±0.80 SD)
1160	Sleep duration (hours per day)	7.21 (±0.94 SD)	7.11 (±1.00 SD)
1170	Getting up in morning (1 = not at all easy, 4 = very easy)	3.13 (±0.74 SD)	3.06 (±0.78 SD)
4570	Friendship satisfaction (1 = extremely happy, 6 = extremely unhappy)	2.15 (±0.69 SD)	2.44 (±0.74 SD)
4559	Family relationship satisfaction (1 = extremely happy, 6 = extremely unhappy)	2.00 (±0.79 SD)	2.49 (±0.88 SD)
1031	Frequency of friend/family visits (1 = almost daily, 7 = no friends/family outside household)	2.76 (±1.07 SD)	2.92 (±1.10 SD)
709	Number in household	2.67 (±1.16 SD)	2.41 (±1.27 SD)
1873	Number of full brothers	1.06 (±1.14 SD)	1.07 (±1.15 SD)
1883	Number of full sisters	0.98 (±1.09 SD)	1.00 (±1.11 SD)
20 127	Neuroticism score (12 = high, 0 = low)	3.39 (±2.97 SD)	4.38 (±3.35 SD)
6160	Leisure/social activities (1 = some regular group activity, 0 = none)	73%	72%
4537	Work/job satisfaction (1 = extremely happy, 7 = not employed)	3.54 (±2.01 SD)	3.69 (±1.96 SD)
1180	Morning/evening person (1 = definitely morning person, 4 = definitely evening person)	2.21 (±0.92 SD)	2.27 (±0.94 SD)
1920	Mood swings	36%	47%
1930	Miserableness	36%	46%
1940	Irritability	25%	32%
1950	Sensitivity/hurt feelings	49%	56%
1960	Fed-up feelings	30%	43%
1970	Nervous feelings	18%	23%
1980	Worrier/anxious feelings	50%	55%
1990	Tense/“highly strung”	13%	18%
2020	Loneliness (often feels lonely)	8%	23%
2000	Worry too long after embarrassment	45%	53%
2010	Suffer from “nerves”	16%	21%
4526	Happiness (1 = extremely happy, 6 = extremely unhappy)	2.36 (±0.66 SD)	2.72 (±0.67 SD)
400	Time to complete pairs matching (deciseconds)	99.01 (±75.32 SD)	102.94 (±101.84 SD)
20 018	Prospective memory test (1 = recall on first attempt, 2 = recall on second attempt)	1.09 (±0.35 SD)	1.10 (±0.36 SD)
1239	Current tobacco smoking (on most or all days)	3%	5%
1249	Past tobacco smoking (1 = most or all days, 4 = never smoked)	2.85 (±1.22 SD)	2.85 (±1.22 SD)
2887	Number of cigarettes previously smoked daily	17.98 (±9.22 SD)	18.34 (±9.67 SD)
2926	Number of unsuccessful stop-smoking attempts	3.16 (±7.66 SD)	3.33 (±9.74 SD)
3476	Difficulty not smoking for 1 day (1 = very easy, 4 = very difficult)	2.84 (±0.95 SD)	2.98 (±0.94 SD)

Using the two variable sets of brain and behavior measurements, we then carried out a bootstrap difference analysis of the collection of target traits in high-SS versus low-SS participants (Efron and Tibshirani 1994). In 1000 bootstrap iterations, we randomly pulled equally sized participant samples to perform a canonical correlation analysis (CCA), in parallel, in high-SS and low-SS individuals (Miller et al. 2016; Wang et al. 2020). In each resampling iteration, this approach estimated the doubly multivariate correspondence between the brain and behavior indicators in each group. The ensuing canonical vectors of the dominant CCA mode indicated the most explanatory demographic associations in a given pull of participants. To directly estimate resample-to-resample effects in group differences, the canonical vectors of behavioral rankings were subtracted elementwise between the low-SS and high-SS participant subsets, recorded, and ultimately aggregated across the 1000 bootstrap datasets.

This analytical tactic allowed propagating the noise of participant sampling variation into the computed uncertainty estimates of group differences in the UK Biobank cohort. Statistically defensible behavioral dimensions were determined by whether the (two-sided) bootstrap confidence interval included zero or not in the 5/95% bootstrap population interval. In a fully multivariate setting, this nonparametric modeling scheme directly quantified the statistical uncertainty of how a UK Biobank trait is differentially linked to brain-behavior correspondence as a function of regular SS.

## Results

Sociodemographic characteristics for high versus low SS individuals are provided in Table 1. Several previous surveys have shown that SS forms an independent and distinct factor of social embeddedness (Cohen and Hoberman 1983; Hawkley et al. 2005; Cyranowski et al. 2013). In our data-set, trait SS was measured by the question “How often are you able to confide in someone close to you?”. Similar items are used in widely embraced SS scales (Cohen and Hoberman 1983; Hawkley et al. 2005; Cyranowski et al. 2013). Such single items were found to highly correlate with full-scale measures of SS (e.g., *r* = 0.88, Hawkley et al. 2005, see Methods for more details). Here, we focused on the comparison between participants with high (“daily or almost daily”) versus low (“less often”) levels of SS.

As a preparatory check, we ascertained the biological meaningfulness of SS as captured in the UK Biobank initiative. Our sample size made it possible to use LD score regression to obtain direct estimates of shared genetic factors between SS and another phenotype of interest (v1.0.0, Bulik-Sullivan et al. 2015). The genome-wide association summary statistics for the SS field were obtained from an open UK Biobank resource (https://github.com/Nealelab/UK_Biobank_GWAS#imputed-v3-phenotypes). The genetic correlations between SS and the full collection of 774 available demographic, lifestyle, and disease phenotypes were then computed using HapMap3 single nucleotide polymorphisms (SNPs) from the LDHUB platform (http://ldsc.broadinstitute.org/ldhub/). After Bonferroni’s correction for multiple comparisons, 52 genetically correlated pairs of phenotypes achieved statistical significance at *P* < 0.05. For example, our SS phenotype shared genetic overlap with happiness (Rg = 0.49 ± 0.05), family relationship satisfaction (Rg = 0.48 ± 0.06), friendship satisfaction (Rg = 0.40 ± 0.05), health satisfaction (Rg = 0.27 ± 0.05), and financial situation satisfaction (Rg = 0.34 ± 0.06). Significant negative genetic correlations were found for recent pain experience (Rg = −0.26 ± 0.04), usual alcohol during meals (Rg = −0.31 ± 0.04), and walking for pleasure (Rg = −0.36 ± 0.05) (see Supplementary Table S1 for full results and *P*-values). These preliminary findings suggest that SS reports from UK Biobank participants capture a heritable biological variation, which is associated with a specific set of driving genetic variants.

Using Bayesian hierarchical modeling, we first explored how interindividual differences in SS are manifested in gray matter volume variation. Our analytical approach was tailored to simultaneously appreciate brain–behavior variation in both distributed large-scale networks and individual regions contained in those brain networks. Anatomical guidance was provided by the Schaefer-Yeo atlas (Schaefer et al. 2018) for cortical regions, and by the Harvard-Oxford atlas (Desikan et al. 2006) for subcortical regions. For clarity, our labeling of brain regions in figures and the discussion will adhere to the conventions given by these used anatomical atlases.

At the network level (Fig. 1), our model inferred the strongest associations with regular SS in the salience network (posterior sigma = 0.080, 10–90% highest posterior density [HPD] = 0.025/0.124) and the limbic network (posterior sigma = 0.069, HPD = 0.002/0.103). A brain network anchored in insula and anterior cingulate cortex was variously referred to as “salience network” (Seeley et al. 2007), “cinguloopercular network” (Dosenbach et al. 2008), “ventral attention network” (Corbetta and Shulman 2002), and “midcingulo-insular network” (Uddin et al. 2019). We recognize that these other nomenclatures have also been used in previous studies referring to this brain system. Henceforth, we refer to this network as the “salience” network for consistency. Smaller volume effects were found for the somatomotor network (posterior sigma = 0.033, HPD = 0.001/0.050), dorsal attention network (posterior sigma = 0.028, HPD = 0.004/0.043), visual network (posterior sigma = 0.025, HPD = 0.001/0.041), DMN (posterior sigma = 0.021, HPD = 0.002/0.034), the frontoparietal control network (posterior sigma = 0.017, HPD = 0.001/0.027), as well as the set of subcortical regions (posterior sigma = 0.023, HPD = 0.005/0.037). Collectively, findings from the higher level of our Bayesian hierarchical model indicate that all examined brain systems showed a degree of brain-behavior associations with SS. However, the regularity of SS in UK Biobank participants was especially attributed to volume variation in the atlas regions belonging to the salience and limbic network.

At the level of regional gray matter variation (Fig. 2), the estimated hierarchical model identified several robust volume effects as a function of regular SS. Positive volume effects in the 10 strongest region-SS associations included the posterior portion of the midcingulate cortex (posterior mean = 0.103, HPD = 0.017/0.178; for more detailed information on regional volume effects, see Supplementary Table S2). Other leading regions with positive volume effects included the anterior temporo-parietal junction (TPJ), close to the supramarginal gyrus, in the right brain (posterior mean = 0.046, HPD = −0.020/0.114) and left brain (posterior mean = 0.037, HPD = −0.025/0.098), the left dorso-lateral prefrontal cortex (dlPFC) corresponding to dorsal middle frontal gyrus (posterior mean = 0.041, HPD = −0.026/0.087), and left anterior/mid cingulate cortex (posterior mean = 0.027, HPD = −0.041/0.088). In contrast, we identified the most relevant negative volume effects in high- versus low-SS individuals in regions of the left anterior insula, extending into parts of the temporal pole and the inferior frontal gyrus (posterior mean = −0.086, HPD = −0.162/0.006; posterior mean = −0.033, HPD = −0.093/0.039). We found additional negative volume effects in the orbitofrontal cortex (OFC) on the left (posterior mean = −0.031, HPD = −0.089/0.029) and right (posterior mean = −0.039, HPD = −0.098/0.030), as well as in the left supplementary motor cortex (posterior mean = −0.073, HPD = −0.013/0.006).

To complement our analysis of SS in brain structure, we next carried out a pattern-learning analysis of the participants’ functional connectivity fingerprints. The collection of 4950 unique connectivity links was submitted to PLS. This analytical approach determined the dominant population mode of covariation in the context of SS (statistically significant at *P* < 0.05, based on nonparametric permutation testing). This mode (Fig. 3) showed that high-SS individuals exhibited increased internetwork connectivity of the salience and limbic networks with dorsal attention and somatomotor networks. In high versus low SS, between-network connectivity among these systems was enhanced, as well as within-network connectivity for systems individually. Conversely, the DMN and, to a smaller extent, the frontoparietal control network exhibited reduced between-network coupling with the visual network for individuals with high SS. As such, being socially well embedded is associated with enhanced functional coupling of brain systems that are widely acknowledged to involve processing aspects in perception–action cycles, rather than higher order processing systems detached from the current sensory environment.

To seek functional understanding of the identified brain correlates of regular SS, we finally conducted a demographic profiling analysis. We computed a CCA that linked a variety of behavioral variables and variation in gray matter volume in our top 10% regions. From the rich UK Biobank population dataset, we selected a wide range of behavioral variables. Our collection covered aspects of physical health, daily habits and lifestyle, substance-use, mental health and wellbeing, and complementary measures of social embeddedness (Table 1).

To recapitulate, this set of leading regions showed the largest effect sizes (as indexed by the mean of the marginal posterior parameter distribution) in our Bayesian hierarchical analysis of SS based on brain structure. Patterns of brain-behavior correspondence were separately computed for high- and low-SS individuals. Our approach thus screened for systematic and robust over- or under-expressions of lifestyle factors in the wider population.

**Figure 4:**
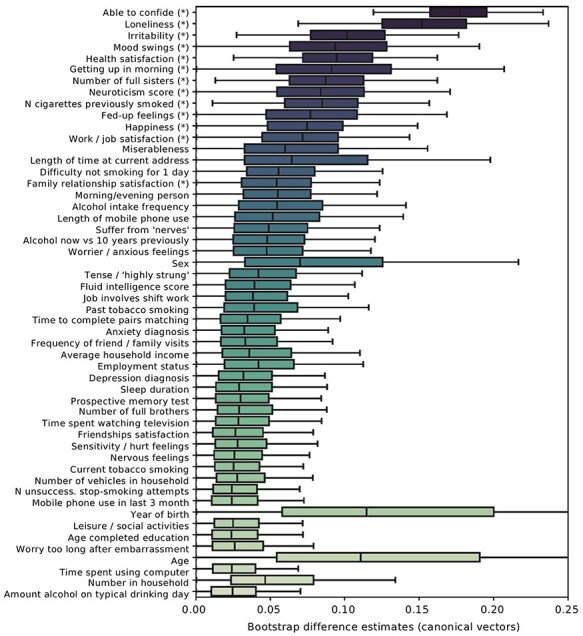
Demographic profiling analysis identifies lifestyle factors related to brain substrates of SS. Multivariate pattern-learning (cf. Methods) was used to explore how the top brain regions (see Fig. 2) are linked to a variety of behavioral indicators in high-SS versus low-SS individuals. Behavioral markers covered domains of mental and physical well-being, lifestyle choices, and social embeddedness. In 1000 bootstrap resampling iterations, our entire pattern-learning pipeline in gray matter volume was repeated separately in the two participant groups: UK Biobank participants who regularly share life experience with close others and those with little such exchange of personal events. The computed differences in brain-behavior associations between both groups (i.e., diverging canonical vector entries) were gathered across the 1000 perturbed redraws of our original dataset to obtain faithful bootstrap intervals at the population level. Note that in the context of the used quantitative modeling framework, age and sex can show relevant effects in conjunction with other behavioral indicators, even if age/sex-related brain variation has been removed in a preceding deconfounding step. The derived estimates of uncertainty directly quantified how group-related deviations vary in the wider population. Asterisks indicate statistical relevance based on excluding zero between the 5/95% quantiles of the bootstrap distribution (cf. Methods, Supplementary Table S3). The boxplot whiskers show the interquartile range (i.e., 25/75% interquartile distance). The highlighted divergences between individuals with weak versus strong SS reveal characteristics of these population strata. Among them were multiple indicators of social embeddedness, health and substance use, as well as factors related to emotional tenseness and distress. An analogous analysis based on functional connectivity did not yield any statistically relevant brain-SS associations. This configuration of brain-behavior differences in high versus low SS speaks to multifaceted manifestations of stress-buffer capacities.

Figure 4 shows the distribution of group differences (i.e., bootstrap difference distributions between canonical vectors, cf. Methods) in high- versus low-SS participants, derived from bootstrapped datasets representing different possible compositions of the population sample. Statistically relevant differences (indicated by asterisks in Fig. 4) between individuals with high versus low regular SS were mainly found for three domains of function. First, highest group differences were found for several measures of social embeddedness that complement the main measure of our study (see also Supplementary Table S3). Specifically, individuals with high versus low SS differed in their brain structural reflections of feelings of loneliness, family relationship satisfaction, and the number of full sisters. Second, group differences were found for associations between brain volume and health- and substance-use factors. Specifically, we observed differences for self-reported health satisfaction, the perceived ease of getting up in the morning, and the severity of previous smoking in those successful at giving up (number of cigarettes previously smoked). As the third collection of differences in brain-behavior associations, individuals with high- versus low-SS differed in their brain structural manifestations of fed-up feelings, levels of irritability, mood swings, neuroticism, as well as happiness and job-satisfaction. As such, group differences were related to several indicators of stress coping behavior and stress buffer.

## Discussion

In challenging times, SS relationships become more important than ever. Social embeddedness is especially crucial for vulnerable populations, such as the elderly (Rodriguez-Laso et al. 2007; Tilvis et al. 2012; Steptoe et al. 2013). Sharing one’s experience and private thoughts with trusted others is critical for coping with stress. Social sciences, public health and other fields agree that physical and mental health hinges on supportive social contact (Holt-Lunstad and Smith 2010; Sutcliffe et al. 2012; Morelli et al. 2017; Dunbar 2018; Holt-Lunstad 2018). Population-scale imaging-genetics cohorts are now opening the door to authentically study the brain basis of everyday lifestyle factors, such as traits of social interaction. Involving ~40 000 UK Biobank participants, we delineated how rich versus poor SS from peers is reflected in brain structure and function in the wider society.

Previous brain-imaging studies in monkeys and humans showed that brain structure is coupled with the size of one’s social group (Lewis et al. 2011; Sallet et al. 2011). By contrast, little is known about the neural implications of quality and closeness of daily social exchanges. We have recently shown that social processes can be differentiated by the degree to which they rely on one of two major brain systems: the default mode and the salience network (Schurz et al. 2021). In the present study, we have focused on the neural reflections of regular SS from close others, and identified a distinctive set of brain regions centering on the salience network. This system was found implicated in more affective, as opposed to cognitive, social processes (Schurz et al. 2021), and more generally in externally oriented processing anchored in the “here-and-now” (Corbetta and Shulman 2002; Dosenbach et al. 2006; Uddin 2015). Collectively, our results therefore pinpoint neural systems that have been rarely described in the context of social embeddedness and friendships (Dunbar 2018; Bzdok and Dunbar 2020).

In brain morphology, we have purpose-designed a Bayesian hierarchical model to test how spatially distributed variation in region volumes of known major brain systems may explain the amount of SS. This analytical strategy uncovered a characteristic pattern of volume increases and decreases in various regions which belong to the salience and limbic networks as a function of SS. Our functional connectivity results show strengthened coupling of the salience and limbic network with other major brain systems for socially well-embedded individuals, including dorsal attention network and somatomotor network.

Especially, the bilateral anterior insula and the anterior/mid cingulate cortex of the salience network have been robustly linked to sharing emotional states that other people experience, as shown by a substantial number of neuroimaging studies (for reviews, see Bzdok et al. 2012; Timmers et al. 2018; Jauniaux et al. 2019; Schurz et al. 2021). For example, a coordinate-based neuroimaging meta-analysis (Timmers et al. 2018) showed that neural activity for socioaffective empathy robustly converges in these areas across different studies and labs. This observation was made across 128 separate neuroimaging experiments conducted in ~3800 participants. Regions of the salience network not only play a role in processing others’ emotions but also in monitoring one’s own internal states (Craig 2009; Singer and Lamm 2009). This 2-fold involvement motivated the view that “shared representations” underlie empathy: our ability to emotionally connect with other people (Preston and de Waal 2002; de Vignemont and Singer 2006; Keysers and Gazzola 2007; Zaki et al. 2016). According to these investigators, the insula and anterior/mid cingulate cortex are probably engaged in similar or identical processes during the direct experience of an affective state and when witnessing someone else in that state (Rütgen et al. 2015; Zhou et al. 2020). Affective states supported by these region’ activity responses are of predominantly negative valence, ranging from pain to basic emotions such as fear, sadness, and disgust (Wicker et al. 2003; Morrison et al. 2004; Singer et al. 2004; Reniers et al. 2014; Toller et al. 2015). Taken together, numerous earlier findings have linked core regions of the salience network to successful sharing of feelings experienced by others (see Menon and Uddin 2010; Uddin 2015).

A possible role of the uncovered brain correlates in understanding others’ emotional states is consistent with a further line of neuroscience research. Tissue lesions in the insula were reported to entail pronounced impairments of understanding others’ emotions (Gu et al. 2012; Leigh et al. 2013; Hillis 2014). For example, acute stroke patients with tissue damage to the anterior insula showed a decline in the capacity to recognize emotional states of other people (Leigh et al. 2013). In these neurological patients, the severity of the deficit in emotion identification was tracked by the lesion volume in the insula. Years after disruption in insular gray matter, the patients still showed deficits in empathic judgments about others (Gu et al. 2012). Similarly, tissue damage in the midcingulate cortex is known to impair reward-contingent processing of contextual information, decision-making, and mounting appropriate reactions (Williams et al. 2004). Hence, causal evidence from several previous studies confirms the relevance of the salience network in social and affective sharing, such as required for SS that people lend to others.

In addition to the anterior insula and midcingulate regions of the salience network, our analyses also identified reliable structural and functional associations of regular SS from close others in the bilateral OFC of the limbic system. These hotspots in the ventromedial prefrontal cortex were repeatedly reported as gray matter correspondences of the sizes of the social circles of humans and monkeys (Sallet et al. 2011; Von Der Heide et al. 2014; Kwak et al. 2018). As part of the limbic system, the medial OFC hosts direct axonal connections with the amygdala (Folloni et al. 2019). The OFC has been linked to processing of reward (Kringelbach 2005) and stimulus–reward associations (Walton et al. 2010), including rewards from social interactions. Brain-imaging studies found that neural activity in the OFC is sensitive to being evaluated by peers in positive or negative ways (Izuma et al. 2008). Orbitofrontal neural activity was also found to track other people’s popularity in real-world social circles (Zerubavel et al. 2015). Individuals with higher gray matter density in the OFC, related to low SS in our study, were shown to have higher social reward dependence (Lebreton et al. 2009). Indeed, previous brain-imaging experiments have reported neural activity responses in these regions when humans think about familiar members of social circles, such as friends opposed to strangers (Krienen et al. 2010).

More broadly, our findings invigorate the distinction between externally oriented and internally oriented brain systems, which may also subserve distinct types of socioaffective cognition. We identified convergent relevance of the anterior insula and the anterior/mid cingulate cortex for the frequent experience of interpersonal sharing. Thus, our findings differ from the regions that are usually reported as candidates for supporting a large number of friends (Lewis et al. 2011; Dunbar 2018; Noonan et al. 2018; Bzdok and Dunbar 2020). In previous studies, it has been hypothesized that the capacity to maintain a circle of regular interaction partners is mainly achieved through more abstract and rational social reflection (Powell et al. 2010; Lewis et al. 2011, 2017), including perspective-taking, the capacity to infer beliefs, behavioral dispositions, and ongoing thoughts of other people (Premack and Woodruff 1978; Frith and Frith 2006; Adolphs 2009; Schurz et al. 2014). In contrast, our study specifically focused on close relationships for sharing personal information that involve mutual trust.

As a view emerging from these present and previous findings, immediate encounters and affective sharing may be a central element of close embeddedness and regular SS. In line with this reasoning, our structural analysis of SS also identified strong effects for bilateral anterior TPJ and left dlPFC. The anterior TPJ was previously implicated in attentional control (Corbetta and Shulman 2002; Decety and Lamm 2007; Krall et al. 2015), as well as in maintaining a “task-set” (Dosenbach et al. 2006) that is believed to support stimulus processing in the present moment. The anterior TPJ has also been described to serve as a “switching device” that is implicated in toggling between functional networks dedicated to externally-oriented processing of the immediate environment and internally oriented processing of self-related mental events (Mars et al. 2012; Bzdok et al. 2013; Kernbach et al. 2018). In social neuroscience experiments, this specific portion of the TPJ has also been reliably linked to distinguishing self- versus other-related representations during affective sharing and appraisal (Silani et al. 2013; Steinbeis et al. 2015; Lamm et al. 2016). Therefore, the anterior TPJ, highlighted bilaterally by our structural analysis of SS, has been associated with control processes, which may be vital for appropriate empathic responding, such as including maintenance of self-others distinction (Silani et al. 2013). Consistently, the dlPFC was also repeatedly implicated in self-other control, such as in regulating emotional self-centeredness (Steinbeis et al. 2015), responses to facial expressions, overcoming racial bias (Cunningham et al. 2004), and overriding prepotent moral judgment (Greene et al. 2004). These collective results encourage the speculation that day-to-day confiding with others can potentially lead to neural changes in systems that implement empathic sharing and response.

Indeed, a seminal longitudinal study on the social brain (Valk et al. 2017) administered daily exercises of emotional sharing to several hundred participants. This regular empathic engagement mediated adaptive increases in gray matter structure, which included the insula, the mid and posterior cingulate cortex, the anterior TPJ, as well as the dlPFC. All of these malleable regions were also highlighted by our population-imaging investigation of regular SS. Neural plasticity gains induced by frequent training indeed coincided with improvements in behavioral assessments of social and emotional skills (Valk et al. 2017). Furthermore, participants also indicated to feel more compassion towards other people who experience pain.

The UK Biobank resource offers deep behavioral and lifestyle characterizations in addition to multimodal neuroimaging measurements. To seize this opportunity, we have implemented pattern-learning techniques to systematically explore multivariate links between gray matter volume for our top brain regions on the one hand, and indicators of social, mental, and physical well-being on the other hand. This demographic enrichment of the brain correlates of SS highlighted associations with emotional tension. Brain manifestations of high versus low SS were robustly linked to indicators related to stress-buffer capacity, resilience, and positive emotion. The identified measures included tendencies for mood swings, fed-up feelings, self-reported irritability and neuroticism, as well as family relationship and job satisfaction. Consistently, our genome-wide analyses revealed shared genetic underpinnings of lacking SS with those of dissatisfaction with one’s social interaction in closer and wider networks, and one’s financial situation. Finally, our brain-behavior analysis and genetic correlation analysis agreed in underscoring a strong link between regular SS and overall happiness.

These results confirm and extend previous research which suggested that social connectedness reduces general levels of psychological distress (Holt-Lunstad 2018; Snyder-Mackler et al. 2020) and anxiety (Finch et al. 1999; St-Jean-Trudel et al. 2009). Research on neuroendocrine systems found that adequate SS can buffer bodily responses to stress, such as those regulated by cortisol hormone pathways through the hypothalamic pituitary adrenal axis (Heinrichs et al. 2003; Ditzen and Heinrichs 2014). In that way, in times of uncertainty and crisis, social connectedness can alleviate negative emotions and worries (Cohen and Syme 1985; Zaki and Williams 2013).

Besides mood-related findings, the demographic profiling and genome-wide analyses also highlighted health- and substance-use related factors in individuals with low versus high SS. Specifically, we found group differences for health satisfaction, difficulties getting up in the morning, as well as smoking behavior and alcohol consumption (the latter only for the genome-wide analysis). These observations dovetail with existing epidemiological work: individuals who consider themselves socially embedded and well-integrated are healthier and live longer than individuals who feel lonely (Cacioppo and Hawkley 2009; Holt-Lunstad and Smith 2010; Luo et al. 2012). The link of social relationships with serious health implications and overall mortality has probably been shown most extensively in older adults. In particular, a meta-analysis of epidemiological studies found these associations to be particularly pronounced in the age range between middle adulthood and age 65 (Holt-Lunstad et al. 2015). The UK Biobank population cohort we studied here shows an age range between 40 and 70 years, which is consistent with this previous work.

Collectively, our brain-imaging and genetics findings point to wide-ranging implications of social connectedness across a variety of lifestyle and health associations. Thus, our population-level evidence reinforces the value of neuroscience insights for developing therapeutic strategies and informing public-health decisions.

## Funding

National Institutes of Health (grant R01AG068563A to D.B.); the Canadian Institutes of Health Research (D.B.); the Natural Sciences and Engineering Research Council of Canada. The project has been made possible with the financial support of Health Canada, through the Canada Brain Research Fund, an innovative partnership between the Government of Canada (through Health Canada) and Brain Canada, and the Montreal Neurological Institute. M.S. was supported by an Erwin Schrödinger Fellowship (FWF-J4009-B27); a Marie Skłodowska-Curie Individual Fellowship (MSCA-IF 844734); the German Research Foundation (SCHN 1481/2-1). The Wellcome Centre for Integrative Neuroimaging is supported by core funding from the Wellcome Trust (203139/Z/16/Z). L.Q.U. was supported by a Gabelli Senior Scholar Award from the University of Miami, and by the National Institute of Mental Health (R01MH107549). P.K. was supported by the German Research Foundation (KA 4412/2-1, KA 4412/4-1, KA 4412/5-1, CRC940/C07). C.L. was supported by the Vienna Science and Technology Fund (WWTF; Project CS11-016) and the Austrian Science Fund (I 3381, P 32686). J.S. was supported by a Wellcome Trust Henry Dale Fellowship (105651/Z/14/Z) and an IDEXLYON IMPULSION 2020 Grant (IDEX/IMP/2020/14). B.B. was supported by the National Science and Engineering Research Council of Canada (NSERC, Discovery-1304413), the Canadian Institutes of Health Research (CIHR, FDN-154298), the Azrieli Center for Autism Research of the Montreal Neurological Institute (ACAR), SickKids Foundation (NI17-039), and the Canada Research Chairs Program. R.B.M. was supported by the Biotechnology and Biological Sciences Research Council (BBSRC) UK (BB/N019814/1), and the Netherlands Organization for Scientific Research NWO (452-13-015). D.B. was supported by the Healthy Brains Healthy Lives initiative (Canada First Research Excellence fund), Google (Teaching Award, Research Award), and by the CIFAR Artificial Intelligence Chairs program (Canada Institute for Advanced Research). For illustrations in our Figures, we used software and images from the Human Connectome Project, WU-Minn Consortium (Principal Investigators: David Van Essen and Kamil Ugurbil; 1U54MH091657) funded by the 16 NIH Institutes and Centers that support the NIH Blueprint for Neuroscience Research; and by the McDonnell Center for Systems Neuroscience at Washington University.

## Notes

Author contributions: Research question, study design, organization of results: D.B., M.S.; methodology, data analysis: D.B.; writing – original draft preparation: all authors; writing – review and editing: all authors. *Conflict of Interest*: Authors declare no competing interests. Data and materials availability: All results of this work are based on data from the UK Biobank, which is openly accessible for researchers. The present analyses were conducted under UK Biobank application number 25163.

## Supplementary Material

SocialSupport_Supplementary_Materials_R1_bhab109

## References

[ref1] Adolphs R. 2009. The social brain: neural basis of social knowledge. Annu Rev Psychol. 60:693–716. 18771388 10.1146/annurev.psych.60.110707.163514 PMC2588649

[ref2] Alcalá-López D, Smallwood J, Jefferies E, Van Overwalle F, Vogeley K, Mars RB, Turetsky BI, Laird AR, Fox PT, Eickhoff SB, et al. 2018. Computing the social brain connectome across systems and states. Cereb Cortex. 28:2207–2232. 28521007 10.1093/cercor/bhx121

[ref3] Alfaro-Almagro F, Jenkinson M, Bangerter NK, Andersson JL, Griffanti L, Douaud G, Sotiropoulos SN, Jbabdi S, Hernandez-Fernandez M, Vallee E, et al. 2018. Image processing and quality control for the first 10,000 brain imaging datasets from UK biobank. Neuroimage. 166:400–424. 29079522 10.1016/j.neuroimage.2017.10.034 PMC5770339

[ref4] Andersson JL, Jenkinson M, Smith S. 2007. Non-linear registration aka Spatial normalisation FMRIB Technical Report TR07JA2. FMRIB Analysis Group of the University of Oxford. www.fmrib.ox.ac.uk/analysis/techrep.

[ref5] Atroszko P, Pianka L, Raczyńska A, Atroszko B, Sęktas M. 2015. Validity and reliability of single-item self-report measures of social support. CER Compar Eur Res. 2015:216.

[ref6] Beckmann CF, Smith SM. 2004. Probabilistic independent component analysis for functional magnetic resonance imaging. IEEE Trans Med Imaging. 23:137–152. 14964560 10.1109/TMI.2003.822821

[ref7] Boss L, Kang D-H, Branson S. 2015. Loneliness and cognitive function in the older adult: a systematic review. Int Psychogeriatr. 27:541–553. 25554219 10.1017/S1041610214002749

[ref8] Bulik-Sullivan BK, Loh P-R, Finucane HK, Ripke S, Yang J, Schizophrenia Working Group of the Psychiatric Genomics Consortium, Patterson N, Daly MJ, Price AL, Neale BM. 2015. LD score regression distinguishes confounding from polygenicity in genome-wide association studies. Nat Genet. 47:291–295. 25642630 10.1038/ng.3211 PMC4495769

[ref9] Bzdok D, Dunbar RIM. 2020. The neurobiology of social distance. Trends Cogn Sci. 24:717–733. 32561254 10.1016/j.tics.2020.05.016 PMC7266757

[ref10] Bzdok D, Eickenberg M, Varoquaux G, Thirion B. 2017. Hierarchical region-network sparsity for high-dimensional inference in brain imaging. Inf Process Med Imaging. 10265:323–335. 29743804 10.1007/978-3-319-59050-9_26 PMC5937695

[ref11] Bzdok D, Floris DL, Marquand AF. 2020. Analysing brain networks in population neuroscience: a case for the Bayesian philosophy. Philos Trans R Soc Lond B Biol Sci. 375:20190661:1–11. 32089111 10.1098/rstb.2019.0661 PMC7061951

[ref12] Bzdok D, Langner R, Schilbach L, Jakobs O, Roski C, Caspers S, Laird AR, Fox PT, Zilles K, Eickhoff SB. 2013. Characterization of the temporo-parietal junction by combining data-driven parcellation, complementary connectivity analyses, and functional decoding. Neuroimage. 81:381–392. 23689016 10.1016/j.neuroimage.2013.05.046 PMC4791053

[ref13] Bzdok D, Schilbach L, Vogeley K, Schneider K, Laird AR, Langner R, Eickhoff SB. 2012. Parsing the neural correlates of moral cognition: ALE meta-analysis on morality, theory of mind, and empathy. Brain Struct Funct. 217:783–796. 22270812 10.1007/s00429-012-0380-y PMC3445793

[ref14] Cacioppo JT, Hawkley LC. 2009. Perceived social isolation and cognition. Trends Cogn Sci. 13:447–54. 19726219 10.1016/j.tics.2009.06.005 PMC2752489

[ref15] Cohen S, Hoberman HM. 1983. Positive events and social supports as buffers of life change Stress1. J Appl Social Pyschol. 13:99–125.

[ref16] Cohen S, Syme SL, editors. 1985. Social support and health. New York: Academic Press.

[ref17] Corbetta M, Shulman GL. 2002. Control of goal-directed and stimulus-driven attention in the brain. Nat Rev Neurosci. 3:201–215. 11994752 10.1038/nrn755

[ref18] Craig AD. 2009. How do you feel — now?. The anterior insula and human awareness. Nat Rev Neurosci. 10:59–70. 10.1038/nrn2555 19096369

[ref19] Cunningham WA, Johnson MK, Raye CL, Chris Gatenby J, Gore JC, Banaji MR. 2004. Separable neural components in the processing of black and white faces. Psychol Sci. 15:806–813. 15563325 10.1111/j.0956-7976.2004.00760.x

[ref20] Cyranowski JM, Zill N, Bode R, Butt Z, Kelly MAR, Pilkonis PA, Salsman JM, Cella D. 2013. Assessing social support, companionship, and distress: National Institute of Health (NIH) toolbox adult social relationship scales. Health Psychol. 32:293–301. 23437856 10.1037/a0028586 PMC3759525

[ref21] Day FR, Ong KK, Perry JRB. 2018. Elucidating the genetic basis of social interaction and isolation. Nat Commun. 9:2457. 29970889 10.1038/s41467-018-04930-1 PMC6030100

[ref22] Decety J, Lamm C. 2007. The role of the right temporoparietal junction in social interaction: how low-level computational processes contribute to meta-cognition. Neuroscientist. 13:580–593. 17911216 10.1177/1073858407304654

[ref23] Desikan RS, Ségonne F, Fischl B, Quinn BT, Dickerson BC, Blacker D, Buckner RL, Dale AM, Maguire RP, Hyman BT, et al. 2006. An automated labeling system for subdividing the human cerebral cortex on MRI scans into gyral based regions of interest. Neuroimage. 31:968–980. 16530430 10.1016/j.neuroimage.2006.01.021

[ref24] de Vignemont F, Singer T. 2006. The empathic brain: how, when and why? Trends Cogn Sci. 10:435–441. 16949331 10.1016/j.tics.2006.08.008

[ref25] Ditzen B, Heinrichs M. 2014. Psychobiology of social support: the social dimension of stress buffering. Restor Neurol Neurosci. 32:149–162. 23603443 10.3233/RNN-139008

[ref26] Dollinger SJ, Malmquist D. 2009. Reliability and validity of single-item self-reports: with special relevance to college students’ alcohol use, religiosity, study, and social life. J Gen Psychol. 136:231–241. 19650519 10.3200/GENP.136.3.231-242

[ref27] Dosenbach NUF, Fair DA, Cohen AL, Schlaggar BL, Petersen SE. 2008. A dual-networks architecture of top-down control. Trends Cogn Sci. 12:99–105. 18262825 10.1016/j.tics.2008.01.001 PMC3632449

[ref28] Dosenbach NUF, Visscher KM, Palmer ED, Miezin FM, Wenger KK, Kang HC, Burgund ED, Grimes AL, Schlaggar BL, Petersen SE. 2006. A core system for the implementation of task sets. Neuron. 50:799–812. 16731517 10.1016/j.neuron.2006.04.031 PMC3621133

[ref29] Dunbar RIM. 1993. Coevolution of neocortical size, group size and language in humans. Behav Brain Sci. 16:681–694.

[ref30] Dunbar RIM. 1998. The social brain hypothesis. Evol Anthropol: Issues News Rev. 6:178–190.

[ref31] Dunbar RIM. 2018. The anatomy of friendship. Trends Cogn Sci. 22:32–51. 29273112 10.1016/j.tics.2017.10.004

[ref32] Efron B. 2012. Large-scale inference: empirical bayes methods for estimation, testing, and prediction. Cambridge (UK): Cambridge University Press.

[ref33] Efron B, Tibshirani RJ. 1994. An introduction to the bootstrap. Boca Raton, FL: Chapman & Hall/CRC.

[ref34] Elovainio M, Hakulinen C, Pulkki-Råback L, Virtanen M, Josefsson K, Jokela M, Vahtera J, Kivimäki M. 2017. Contribution of risk factors to excess mortality in isolated and lonely individuals: an analysis of data from the UK biobank cohort study. Lancet Public Health. 2:e260–e266. 28626828 10.1016/S2468-2667(17)30075-0 PMC5463031

[ref35] Elwert F, Christakis NA. 2008. The effect of widowhood on mortality by the causes of death of both spouses. Am J Public Health. 98:2092–2098. 18511733 10.2105/AJPH.2007.114348 PMC2636447

[ref36] Evolahti A, Hultcrantz M, Collins A. 2006. Women’s work stress and cortisol levels: a longitudinal study of the association between the psychosocial work environment and serum cortisol. J Psychosom Res. 61:645–652. 17084142 10.1016/j.jpsychores.2006.07.022

[ref37] Finch JF, Okun MA, Pool GJ, Ruehlman LS. 1999. A comparison of the influence of conflictual and supportive social interactions on psychological distress. J Pers. 67:581–621. 10444852 10.1111/1467-6494.00066

[ref38] Folloni D, Sallet J, Khrapitchev AA, Sibson N, Verhagen L, Mars RB. 2019. Dichotomous organization of amygdala/temporal-prefrontal bundles in both humans and monkeys. Elife. 8:e47175. 1–23. 10.7554/eLife.47175 PMC6831033 31689177

[ref39] Fowler JH, Christakis NA. 2008. Dynamic spread of happiness in a large social network: longitudinal analysis over 20 years in the Framingham heart study. BMJ. 337:a2338. 19056788 10.1136/bmj.a2338 PMC2600606

[ref40] Frith CD, Frith U. 2006. The neural basis of mentalizing. Neuron. 50:531–534. 16701204 10.1016/j.neuron.2006.05.001

[ref42] Gelman A, Carlin JB, Stern HS, Rubin DB. 2014. Bayesian data analysis. Vol 2. Boca Raton, FL: Chapman & Hall/CRC.

[ref43] Granovetter MS. 1973. The strength of weak ties. Am J Sociol., 78:1360–1380.

[ref44] Granovetter MS. 1983. The strength of weak ties: a network theory revisited. Sociol Theor. 1:201–233.

[ref45] Greene JD, Nystrom LE, Engell AD, Darley JM, Cohen JD. 2004. The neural bases of cognitive conflict and control in moral judgment. Neuron. 44:389–400. 15473975 10.1016/j.neuron.2004.09.027

[ref46] Griffanti L, Salimi-Khorshidi G, Beckmann CF, Auerbach EJ, Douaud G, Sexton CE, Zsoldos E, Ebmeier KP, Filippini N, Mackay CE, et al. 2014. ICA-based artefact removal and accelerated fMRI acquisition for improved resting state network imaging. Neuroimage. 95:232–247. 24657355 10.1016/j.neuroimage.2014.03.034 PMC4154346

[ref47] Gu X, Gao Z, Wang X, Liu X, Knight RT, Hof PR, Fan J. 2012. Anterior insular cortex is necessary for empathetic pain perception. Brain. 135:2726–2735. 22961548 10.1093/brain/aws199 PMC3437027

[ref48] Hall JA. 2019. How many hours does it take to make a friend? J Soc Pers Relat. 36:1278–1296.

[ref49] Hawkley LC, Browne MW, Cacioppo JT. 2005. How can I connect with thee? Let me count the ways. Psychol Sci. 16:798–804. 16181443 10.1111/j.1467-9280.2005.01617.x

[ref50] Hawkley LC, Burleson MH, Berntson GG, Cacioppo JT. 2003. Loneliness in everyday life: cardiovascular activity, psychosocial context, and health behaviors. J Pers Soc Psychol. 85:105–120. 12872887 10.1037/0022-3514.85.1.105

[ref51] Heinrichs M, Baumgartner T, Kirschbaum C, Ehlert U. 2003. Social support and oxytocin interact to suppress cortisol and subjective responses to psychosocial stress. Biol Psychiatry. 54:1389–1398. 14675803 10.1016/s0006-3223(03)00465-7

[ref52] Hillis AE. 2014. Inability to empathize: brain lesions that disrupt sharing and understanding another’s emotions. Brain. 137:981–997. 24293265 10.1093/brain/awt317 PMC3959550

[ref53] Holt-Lunstad J. 2018. Why social relationships are important for physical health: a systems approach to understanding and modifying risk and protection. Annu Rev Psychol. 69:437–458. 29035688 10.1146/annurev-psych-122216-011902

[ref54] Holt-Lunstad J, Smith T. 2010. Social relationships and mortality risk: a meta-analytic review. PLoS Med. 7(7). 10.1371/journal.pmed.1000316 PMC2910600 20668659

[ref55] Holt-Lunstad J, Smith TB, Baker M, Harris T, Stephenson D. 2015. Loneliness and social isolation as risk factors for mortality: a meta-analytic review. Perspect Psychol Sci. 10:227–237. 25910392 10.1177/1745691614568352

[ref56] Izuma K, Saito DN, Sadato N. 2008. Processing of social and monetary rewards in the human striatum. Neuron. 58:284–294. 18439412 10.1016/j.neuron.2008.03.020

[ref57] Jauniaux J, Khatibi A, Rainville P, Jackson PL. 2019. A meta-analysis of neuroimaging studies on pain empathy: investigating the role of visual information and observers’ perspective. Soc Cogn Affect Neurosci. 14:789–813. 31393982 10.1093/scan/nsz055 PMC6847411

[ref58] Jenkinson M, Bannister P, Brady M, Smith S. 2002. Improved optimization for the robust and accurate linear registration and motion correction of brain images. Neuroimage. 17:825–841. 12377157 10.1016/s1053-8119(02)91132-8

[ref59] Jenkinson M, Smith S. 2001. A global optimisation method for robust affine registration of brain images. Med Image Anal. 5:143–156. 11516708 10.1016/s1361-8415(01)00036-6

[ref60] Kardos P, Leidner B, Pléh C, Soltész P, Unoka Z. 2017. Empathic people have more friends: empathic abilities predict social network size and position in social network predicts empathic efforts. Soc Networks. 50:1–5.

[ref61] Kernbach JM, Yeo BTT, Smallwood J, Margulies DS, Thiebaut de Schotten M, Walter H, Sabuncu MR, Holmes AJ, Gramfort A, Varoquaux G, et al. 2018. Subspecialization within default mode nodes characterized in 10,000 UK biobank participants. Proc Natl Acad Sci USA. 115:12295–12300. 30420501 10.1073/pnas.1804876115 PMC6275484

[ref62] Keysers C, Gazzola V. 2007. Integrating simulation and theory of mind: from self to social cognition. Trends Cogn Sci. 11:194–196. 17344090 10.1016/j.tics.2007.02.002

[ref63] Kiesow H, Dunbar RIM, Kable JW, Kalenscher T, Vogeley K, Schilbach L, Marquand AF, Wiecki TV, Bzdok D. 2020. 10,000 social brains: sex differentiation in human brain anatomy. Sci Adv. 6:eaaz1170. 1–12. 32206722 10.1126/sciadv.aaz1170 PMC7080454

[ref64] Kim DA, Benjamin EJ, Fowler JH, Christakis NA. 2016. Social connectedness is associated with fibrinogen level in a human social network. Proc Biol Sci. 283:20160958. 1–7. 27559060 10.1098/rspb.2016.0958 PMC5013792

[ref65] Krall SC, Rottschy C, Oberwelland E, Bzdok D, Fox PT, Eickhoff SB, Fink GR, Konrad K. 2015. The role of the right temporoparietal junction in attention and social interaction as revealed by ALE meta-analysis. Brain Struct Funct. 220:587–604. 24915964 10.1007/s00429-014-0803-z PMC4791048

[ref66] Krienen FM, Tu P-C, Buckner RL. 2010. Clan mentality: evidence that the medial prefrontal cortex responds to close others. J Neurosci. 30:13906–13915. 20943931 10.1523/JNEUROSCI.2180-10.2010 PMC2989424

[ref67] Kringelbach ML. 2005. The human orbitofrontal cortex: linking reward to hedonic experience. Nat Rev Neurosci. 6:691–702. 16136173 10.1038/nrn1747

[ref68] Kuiper JS, Zuidersma M, Oude Voshaar RC, Zuidema SU, van den Heuvel ER, Stolk RP, Smidt N. 2015. Social relationships and risk of dementia: a systematic review and meta-analysis of longitudinal cohort studies. Ageing Res Rev. 22:39–57. 25956016 10.1016/j.arr.2015.04.006

[ref69] Kurina LM, Knutson KL, Hawkley LC, Cacioppo JT, Lauderdale DS, Ober C. 2011. Loneliness is associated with sleep fragmentation in a communal society. Sleep. 34:1519–1526. 22043123 10.5665/sleep.1390 PMC3198207

[ref70] Kwak S, Joo W-T, Youm Y, Chey J. 2018. Social brain volume is associated with in-degree social network size among older adults. Proc Biol Sci. 285:20172708. 1–10. 29367402 10.1098/rspb.2017.2708 PMC5805955

[ref71] Lamm C, Bukowski H, Silani G. 2016. From shared to distinct self-other representations in empathy: evidence from neurotypical function and socio-cognitive disorders. Philos Trans R Soc Lond B Biol Sci. 371:20150083. 1–7. 26644601 10.1098/rstb.2015.0083 PMC4685528

[ref72] Lebreton M, Barnes A, Miettunen J, Peltonen L, Ridler K, Veijola J, Tanskanen P, Suckling J, Jarvelin M-R, Jones PB, et al. 2009. The brain structural disposition to social interaction. Eur J Neurosci. 29:2247–2252. 19490022 10.1111/j.1460-9568.2009.06782.x

[ref73] Leigh R, Oishi K, Hsu J, Lindquist M, Gottesman RF, Jarso S, Crainiceanu C, Mori S, Hillis AE. 2013. Acute lesions that impair affective empathy. Brain. 136:2539–2549. 23824490 10.1093/brain/awt177 PMC3722353

[ref74] Lelkes O. 2010. Social Participation and Social Isolation. In: Atkinson AB, Marlier E, editors. Income and Living Conditions in Europe. Luxembourg: Eurostat, European Commission. pp. 217–231.

[ref75] Le V, Arayasirikul S, Chen Y-H, Jin H, Wilson EC. 2016. Types of social support and parental acceptance among transfemale youth and their impact on mental health, sexual debut, history of sex work and condomless anal intercourse. J Int AIDS Soc. 19:20781. 1–6. 27431467 10.7448/IAS.19.3.20781 PMC4949317

[ref76] Lewis PA, Birch A, Hall A, Dunbar RIM. 2017. Higher order intentionality tasks are cognitively more demanding. Soc Cogn Affect Neurosci. 12:1063–1071. 28338962 10.1093/scan/nsx034 PMC5490680

[ref77] Lewis PA, Rezaie R, Brown R, Roberts N, Dunbar RIM. 2011. Ventromedial prefrontal volume predicts understanding of others and social network size. Neuroimage. 57:1624–1629. 21616156 10.1016/j.neuroimage.2011.05.030 PMC7614375

[ref78] Luhmann M, Hawkley LC. 2016. Age differences in loneliness from late adolescence to oldest old age. Dev Psychol. 52:943–959. 27148782 10.1037/dev0000117 PMC8015413

[ref79] Luo Y, Hawkley LC, Waite LJ, Cacioppo JT. 2012. Loneliness, health, and mortality in old age: a national longitudinal study. Soc Sci Med. 74:907–914. 22326307 10.1016/j.socscimed.2011.11.028 PMC3303190

[ref80] Mars RB, Sallet J, Schüffelgen U, Jbabdi S, Toni I, Rushworth MFS. 2012. Connectivity-based subdivisions of the human right “Temporoparietal junction area”: evidence for different areas participating in different cortical networks. Cereb Cortex. 22:1894–1903. 21955921 10.1093/cercor/bhr268

[ref81] Mashek D, Cannaday LW, Tangney JP. 2007. Inclusion of community in self scale: a single-item pictorial measure of community connectedness. J Community Psychol. 35:257–275.

[ref82] Menon V, Uddin LQ. 2010. Saliency, switching, attention and control: a network model of insula function. Brain Struct Funct. 214:655–667. 20512370 10.1007/s00429-010-0262-0 PMC2899886

[ref83] Miller KL, Alfaro-Almagro F, Bangerter NK, Thomas DL, Yacoub E, Xu J, Bartsch AJ, Jbabdi S, Sotiropoulos SN, Andersson JL, et al. 2016. Multimodal population brain imaging in the UK Biobank prospective epidemiological study. Nat Neurosci. 19:1523–1536. 27643430 10.1038/nn.4393 PMC5086094

[ref84] Morelli SA, Ong DC, Makati R, Jackson MO, Zaki J. 2017. Empathy and well-being correlate with centrality in different social networks. Proc Natl Acad Sci USA. 114:9843–9847. 28851835 10.1073/pnas.1702155114 PMC5604000

[ref85] Morrison I, Lloyd D, Di Pellegrino G, Roberts N. 2004. Vicarious responses to pain in anterior cingulate cortex: is empathy a multisensory issue? Cogn Affect Behav Neurosci. 4:270–278. 15460933 10.3758/cabn.4.2.270

[ref86] Noonan MP, Mars RB, Sallet J, Dunbar RIM, Fellows LK. 2018. The structural and functional brain networks that support human social networks. Behav Brain Res. 355:12–23. 29471028 10.1016/j.bbr.2018.02.019 PMC6152579

[ref87] Ong AD, Uchino BN, Wethington E. 2016. Loneliness and health in older adults: a mini-review and synthesis. Gerontology. 62:443–449. 26539997 10.1159/000441651 PMC6162046

[ref88] Powell JL, Lewis PA, Dunbar RIM, García-Fiñana M, Roberts N. 2010. Orbital prefrontal cortex volume correlates with social cognitive competence. Neuropsychologia. 48:3554–3562. 20713074 10.1016/j.neuropsychologia.2010.08.004

[ref89] Premack D, Woodruff G. 1978. Does the chimpanzee have a theory of mind? Behav Brain Sci. 1:515–526.

[ref90] Pressman SD, Cohen S, Miller GE, Barkin A, Rabin BS, Treanor JJ. 2005. Loneliness, social network size, and immune response to influenza vaccination in college freshmen. Health Psychol. 24:297–306. 15898866 10.1037/0278-6133.24.3.297

[ref91] Preston SD, de Waal FBM. 2002. Empathy: its ultimate and proximate bases. Behav Brain Sci. 25:1–20 discussion 20–71. 12625087 10.1017/s0140525x02000018

[ref92] Reblin M, Uchino BN. 2008. Social and emotional support and its implication for health. Curr Opin Psychiatry. 21:201–205. 18332671 10.1097/YCO.0b013e3282f3ad89 PMC2729718

[ref93] Redcay E, Schilbach L. 2019. Using second-person neuroscience to elucidate the mechanisms of social interaction. Nat Rev Neurosci. 20:495–505. 31138910 10.1038/s41583-019-0179-4 PMC6997943

[ref94] Reniers RLEP, Renate LE, Völlm BA, Elliott R, Corcoran R. 2014. Empathy, ToM, and self–other differentiation: an fMRI study of internal states. Soc Neurosci. 9:50–62. 24294841 10.1080/17470919.2013.861360

[ref95] Rodriguez-Laso A, Zunzunegui MV, Otero A. 2007. The effect of social relationships on survival in elderly residents of a southern European community: a cohort study. BMC Geriatr. 7:19. 17678536 10.1186/1471-2318-7-19 PMC2042977

[ref96] Rütgen M, Seidel E-M, Silani G, Riečanský I, Hummer A, Windischberger C, Petrovic P, Lamm C. 2015. Placebo analgesia and its opioidergic regulation suggest that empathy for pain is grounded in self pain. Proc Natl Acad Sci USA. 112:E5638–E5646. 26417092 10.1073/pnas.1511269112 PMC4611649

[ref97] Sallet J, Mars RB, Noonan MP, Andersson JL, O’Reilly JX, Jbabdi S, Croxson PL, Jenkinson M, Miller KL, Rushworth MFS. 2011. Social network size affects neural circuits in macaques. Science. 334:697–700. 22053054 10.1126/science.1210027

[ref98] Salvatier J, Wiecki TV, Fonnesbeck C. 2016. Probabilistic programming in python using PyMC3. PeerJ Computer Science. 2:e55. 1–24.

[ref99] Sasaki Y, Aida J, Tsuji T, Koyama S, Tsuboya T, Saito T, Kondo K, Kawachi I. 2019. Pre-disaster social support is protective for onset of post-disaster depression: prospective study from the Great East Japan Earthquake & Tsunami. Sci Rep. 9:19427. 1–10. 31857658 10.1038/s41598-019-55953-7 PMC6923367

[ref100] Schaefer A, Kong R, Gordon EM, Laumann TO, Zuo X-N, Holmes AJ, Eickhoff SB, Yeo BTT. 2018. Local-global parcellation of the human cerebral cortex from intrinsic functional connectivity MRI. Cereb Cortex. 28:3095–3114. 28981612 10.1093/cercor/bhx179 PMC6095216

[ref101] Schurz M, Radua J, Aichhorn M, Richlan F, Perner J. 2014. Fractionating theory of mind: a meta-analysis of functional brain imaging studies. Neurosci Biobehav Rev. 42:9–34. 24486722 10.1016/j.neubiorev.2014.01.009

[ref102] Schurz M, Radua J, Tholen Matthias G, Margulies DG, Mars RB, Sallet J, Kanske P. 2021. Towards a hierarchical model of social cognition: a neuroimaging meta-analysis and integrative review on empathy and theory of mind. Psychol Bull. 147:293–327. 33151703 10.1037/bul0000303

[ref103] Seeley WW, Menon V, Schatzberg AF, Keller J, Glover GH, Kenna H, Reiss AL, Greicius MD. 2007. Dissociable intrinsic connectivity networks for salience processing and executive control. J Neurosci. 27:2349–2356. 17329432 10.1523/JNEUROSCI.5587-06.2007 PMC2680293

[ref104] Silani G, Lamm C, Ruff CC, Singer T. 2013. Right Supramarginal gyrus is crucial to overcome emotional egocentricity bias in social judgments. J Neurosci. 33:15466–15476. 24068815 10.1523/JNEUROSCI.1488-13.2013 PMC6618458

[ref105] Singer T, Lamm C. 2009. The social neuroscience of empathy. Ann N Y Acad Sci. 1156:81–96. 19338504 10.1111/j.1749-6632.2009.04418.x

[ref106] Singer T, Seymour B, O’Doherty J, Kaube H, Dolan RJ, Frith CD. 2004. Empathy for pain involves the affective but not sensory components of pain. Science. 303:1157–1162. 14976305 10.1126/science.1093535

[ref107] Smith SM. 2002. Fast robust automated brain extraction. Hum Brain Mapp. 17:143–155. 12391568 10.1002/hbm.10062 PMC6871816

[ref108] Smith SM, Zhang Y, Jenkinson M, Chen J, Matthews PM, Federico A, De Stefano N. 2002. Accurate, robust, and automated longitudinal and cross-sectional brain change analysis. Neuroimage. 17:479–489. 12482100 10.1006/nimg.2002.1040

[ref109] Snyder-Mackler N, Burger JR, Gaydosh L, Belsky DW, Noppert GA, Campos FA, Bartolomucci A, Yang YC, Aiello AE, O’Rand A, et al. 2020. Social determinants of health and survival in humans and other animals. Science. 368:eaax9553. 1–12 32439765 10.1126/science.aax9553 PMC7398600

[ref110] Spreng RN, Dimas E, Mwilambwe-Tshilobo L, Dagher A, Koellinger P, Nave G, Ong A, Kernbach JM, Wiecki TV, Ge T, et al. 2020. The default network of the human brain is associated with perceived social isolation. Nat Commun. 11:1–11. 33319780 10.1038/s41467-020-20039-w PMC7738683

[ref111] Spunt RP, Adolphs R. 2017. A new look at domain specificity: insights from social neuroscience. Nat Rev Neurosci. 18:559–567. 28680161 10.1038/nrn.2017.76

[ref112] Steinbeis N, Bernhardt BC, Singer T. 2015. Age-related differences in function and structure of rSMG and reduced functional connectivity with DLPFC explains heightened emotional egocentricity bias in childhood. Soc Cogn Affect Neurosci. 10:302–310. 24771281 10.1093/scan/nsu057 PMC4321629

[ref113] Steptoe A, Shankar A, Demakakos P, Wardle J. 2013. Social isolation, loneliness, and all-cause mortality in older men and women. Proc Natl Acad Sci USA. 110:5797–5801. 23530191 10.1073/pnas.1219686110 PMC3625264

[ref114] St-Jean-Trudel E, Guay S, Marchand A. 2009. The relationship between social support, psychological stress and the risk of developing anxiety disorders in men and women: results of a national study. Can J Publ Health Revue canadienne de sante publique. 100:148–152. 10.1007/BF03405526 PMC6974214 19839294

[ref115] Sutcliffe A, Dunbar R, Binder J, Arrow H. 2012. Relationships and the social brain: integrating psychological and evolutionary perspectives. Br J Psychol. 103:149–168. 22506741 10.1111/j.2044-8295.2011.02061.x

[ref116] Tilvis RS, Routasalo P, Karppinen H, Strandberg TE, Kautiainen H, Pitkala KH. 2012. Social isolation, social activity and loneliness as survival indicators in old age; a nationwide survey with a 7-year follow-up. Eur Geriatr Med. 3:18–22.

[ref117] Timmers I, Park AL, Fischer MD, Kronman CA, Heathcote LC, Hernandez JM, Simons LE. 2018. Is empathy for pain unique in its neural correlates? A meta-analysis of neuroimaging studies of empathy. Front Behav Neurosci. 12:289. 30542272 10.3389/fnbeh.2018.00289 PMC6277791

[ref118] Toi M, Batson DC. 1982. More evidence that empathy is a source of altruistic motivation. J Pers Soc Psychol. 43:281–292.

[ref119] Toller G, Adhimoolam B, Grunwald T, Huppertz H-J, Kurthen M, Rankin KP, Jokeit H. 2015. Right mesial temporal lobe epilepsy impairs empathy-related brain responses to dynamic fearful faces. J Neurol. 262:729–741. 25572160 10.1007/s00415-014-7622-2

[ref120] Uddin LQ. 2015. Salience processing and insular cortical function and dysfunction. Nat Rev Neurosci. 16:55–61. 25406711 10.1038/nrn3857

[ref121] Uddin LQ, Yeo BTT, Spreng RN. 2019. Towards a universal taxonomy of macro-scale functional human brain networks. Brain Topogr. 32:926–942. 31707621 10.1007/s10548-019-00744-6 PMC7325607

[ref122] Valk SL, Bernhardt BC, Trautwein F-M, Böckler A, Kanske P, Guizard N, Collins DL, Singer T. 2017. Structural plasticity of the social brain: differential change after socio-affective and cognitive mental training. Sci Adv. 3:e1700489. 1–11 28983507 10.1126/sciadv.1700489 PMC5627980

[ref123] Von Der Heide R, Vyas G, Olson IR. 2014. The social network-network: size is predicted by brain structure and function in the amygdala and paralimbic regions. Soc Cogn Affect Neurosci. 9:1962–1972. 24493846 10.1093/scan/nsu009 PMC4249478

[ref124] Walton ME, Behrens TEJ, Buckley MJ, Rudebeck PH, Rushworth MFS. 2010. Separable learning systems in the macaque brain and the role of orbitofrontal cortex in contingent learning. Neuron. 65:927–939. 20346766 10.1016/j.neuron.2010.02.027 PMC3566584

[ref125] Wang H-T, Smallwood J, Mourao-Miranda J, Xia CH, Satterthwaite TD, Bassett DS, Bzdok D. 2020. Finding the needle in a high-dimensional haystack: canonical correlation analysis for neuroscientists. Neuroimage. 216:116745. 1–14 32278095 10.1016/j.neuroimage.2020.116745

[ref126] Wicker B, Keysers C, Plailly J, Royet JP, Gallese V, Rizzolatti G. 2003. Both of us disgusted in my insula: the common neural basis of seeing and feeling disgust. Neuron. 40:655–664. 14642287 10.1016/s0896-6273(03)00679-2

[ref127] Williams ZM, Bush G, Rauch SL, Cosgrove GR, Eskandar EN. 2004. Human anterior cingulate neurons and the integration of monetary reward with motor responses. Nat Neurosci. 7:1370–1375. 15558064 10.1038/nn1354

[ref128] Zaki J, Wager TD, Singer T, Keysers C, Gazzola V. 2016. The anatomy of suffering: understanding the relationship between nociceptive and empathic pain. Trends Cogn Sci. 20:249–259. 26944221 10.1016/j.tics.2016.02.003 PMC5521249

[ref129] Zaki J, Williams WC. 2013. Interpersonal emotion regulation. Emotion. 13:803–810. 24098929 10.1037/a0033839

[ref130] Zerubavel N, Bearman PS, Weber J, Ochsner KN. 2015. Neural mechanisms tracking popularity in real-world social networks. Proc Natl Acad Sci USA. 112:15072–15077. 26598684 10.1073/pnas.1511477112 PMC4679039

[ref131] Zhang Y, Brady M, Smith S. 2001. Segmentation of brain MR images through a hidden Markov random field model and the expectation-maximization algorithm. IEEE Trans Med Imaging. 20:45–57. 11293691 10.1109/42.906424

[ref132] Zhou F, Li J, Zhao W, Xu L, Zheng X, Fu M, Yao S. 2020. Emotional contagion of pain across different social cues shares common and process-specific neural representations. eLife. 2020 9:e56929. 32894226 10.7554/eLife.56929 PMC7505665

