# Supplementary material for: Variability in Brain Structure and Function Reflects Lack of Peer Support
Source: Cereb Cortex. 2021 May 13;31(10):4612–27. doi: 10.1093/cercor/bhab109 (PMC8408465; doi:10.1093/cercor/bhab109)

**Supplementary Tables**

**Table S1.** Genetic correlations.

**
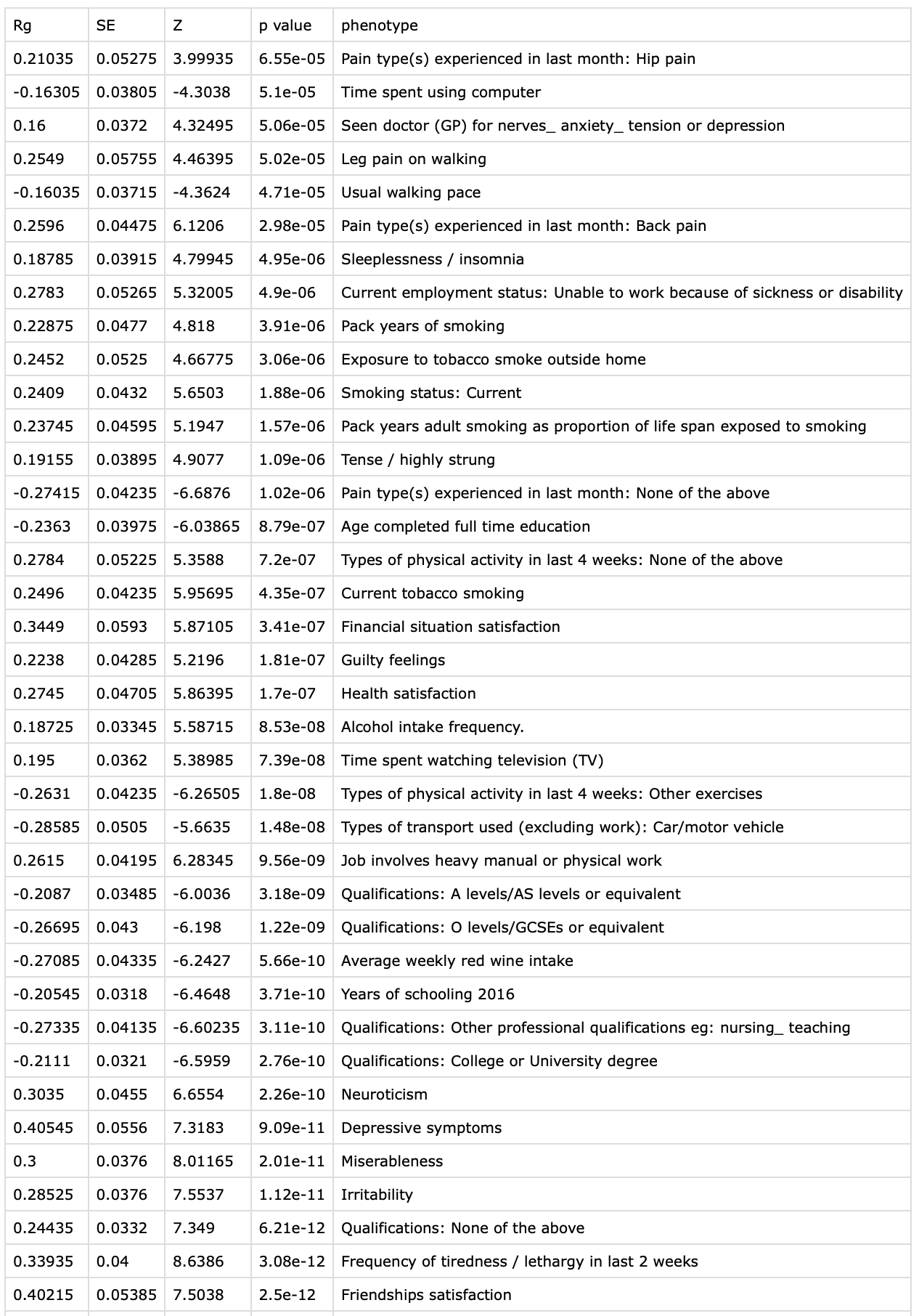
**

**Table S1 continued.** Genetic correlations.


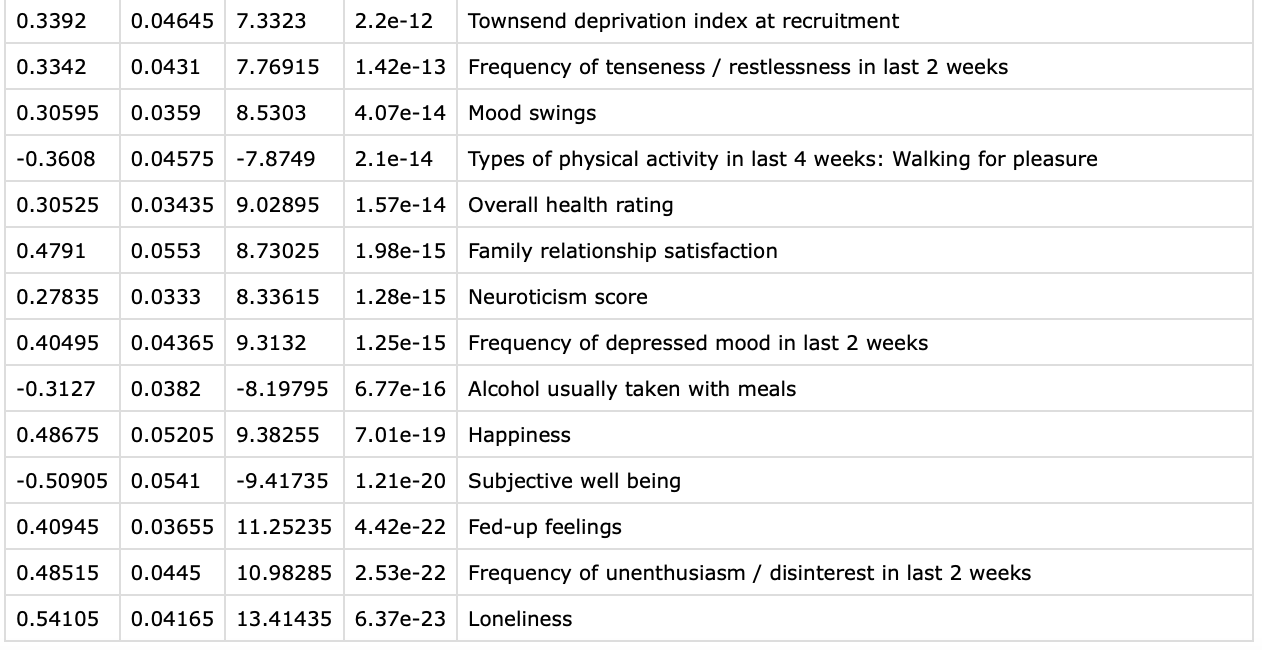

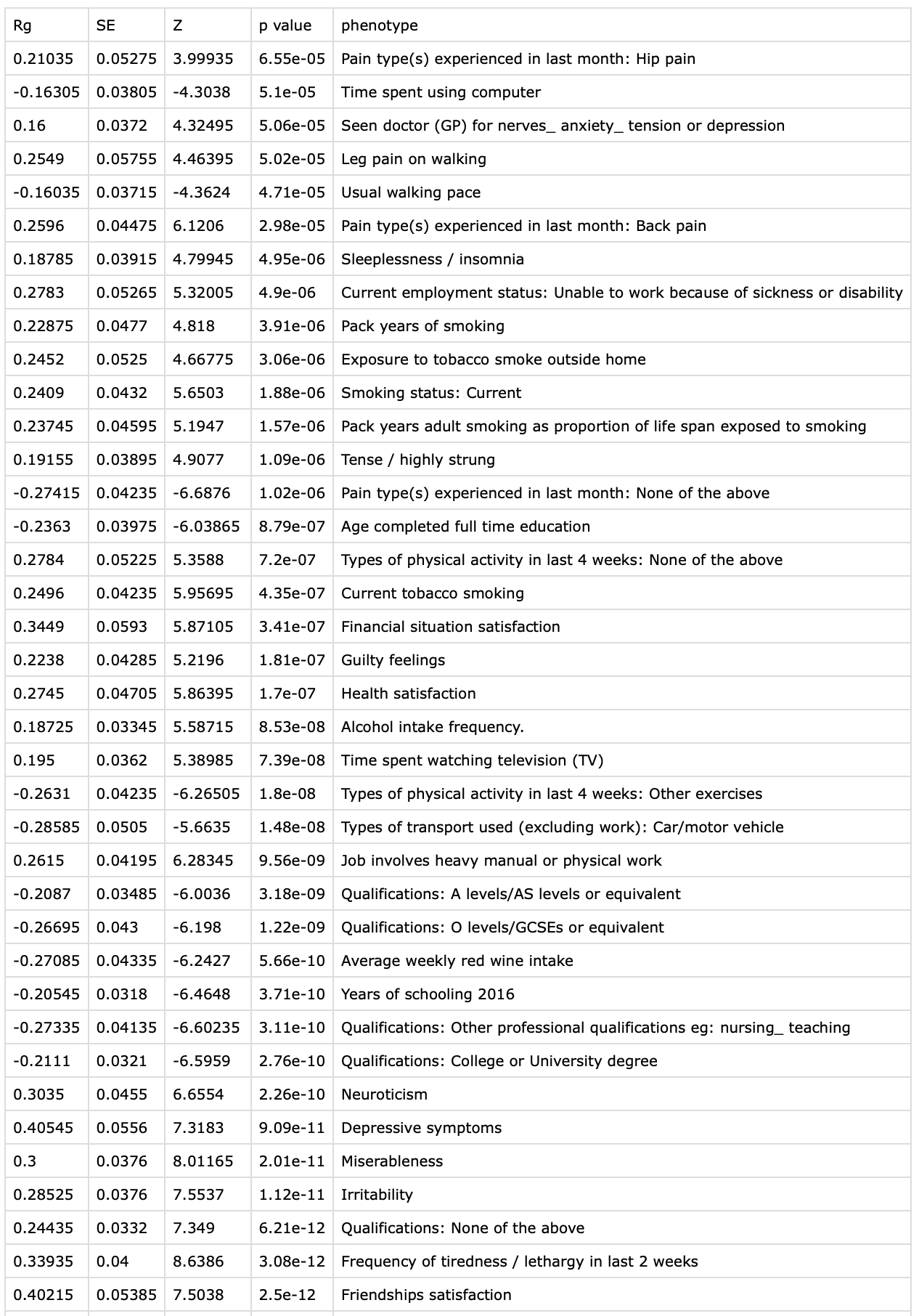


Table S2. List of the identified 35 regions with volume effects for which the HPD mainly fell on one side of the sign (i.e. at least ⅔ of posterior probability mass indicates positive or negative effect).

| Label | Mean | HPD_10% | HPD_90% |
| --- | --- | --- | --- |
| **RH_SalVentAttn_Med_1** | 0.103 | 0.017 | 0.178 |
| **LH_SalVentAttn_FrOperIns_2** | -0.086 | -0.162 | 0.006 |
| **LH_SalVentAttn_Med_3** | -0.073 | -0.13 | 0.006 |
| **RH_SalVentAttn_TempOccPar_2** | 0.046 | -0.02 | 0.114 |
| **LH_SalVentAttn_PFCl_1** | 0.041 | -0.026 | 0.087 |
| **RH_Limbic_OFC_1** | -0.039 | -0.098 | 0.03 |
| **LH_SalVentAttn_ParOper_1** | 0.037 | -0.025 | 0.098 |
| **LH_SalVentAttn_FrOperIns_1** | -0.033 | -0.093 | 0.039 |
| **LH_Limbic_OFC_1** | -0.031 | -0.089 | 0.029 |
| **LH_SalVentAttn_Med_1** | 0.027 | -0.041 | 0.088 |
| RH_SalVentAttn_TempOccPar_1 | -0.027 | -0.108 | 0.026 |
| LH_SomMot_6 | -0.024 | -0.066 | 0.017 |
| RH_Limbic_TempPole_1 | -0.023 | -0.09 | 0.04 |
| Ventral Striatum (left) | -0.023 | -0.046 | 0.008 |
| RH_DorsAttn_Post_3 | 0.02 | -0.016 | 0.05 |
| LH_SomMot_4 | -0.02 | -0.058 | 0.017 |
| Caudate (right) | 0.019 | -0.007 | 0.046 |
| Hippocampus (left) | 0.019 | -0.015 | 0.04 |
| RH_DorsAttn_FEF_1 | 0.019 | -0.016 | 0.05 |
| Hippocampus (right) | -0.019 | -0.04 | 0.01 |
| LH_Limbic_TempPole_1 | 0.018 | -0.038 | 0.085 |
| Pallidum (left) | 0.018 | -0.005 | 0.039 |
| RH_SomMot_2 | 0.017 | -0.023 | 0.05 |
| LH_Vis_8 | -0.016 | -0.047 | 0.015 |
| LH_DorsAttn_PrCv_1 | 0.014 | -0.018 | 0.045 |
| Thalamus (right) | -0.014 | -0.034 | 0.015 |
| RH_SomMot_4 | -0.013 | -0.05 | 0.022 |
| LH_Default_pCunPCC_1 | 0.011 | -0.017 | 0.036 |
| LH_DorsAttn_Post_2 | 0.011 | -0.016 | 0.05 |
| LH_Default_Temp_2 | -0.011 | -0.033 | 0.016 |
| Amygdala (right) | -0.01 | -0.035 | 0.011 |
| RH_Vis_8 | -0.01 | -0.041 | 0.017 |
| Brain-Stem | 0.009 | -0.013 | 0.03 |
| Ventral Striatum (right) | -0.008 | -0.036 | 0.011 |
| LH_Default_pCunPCC_2 | 0.006 | -0.015 | 0.034 |

*Top 10 regions are highlighted in bold.*

Table S3. Demographic profiling results for sMRI in detail.


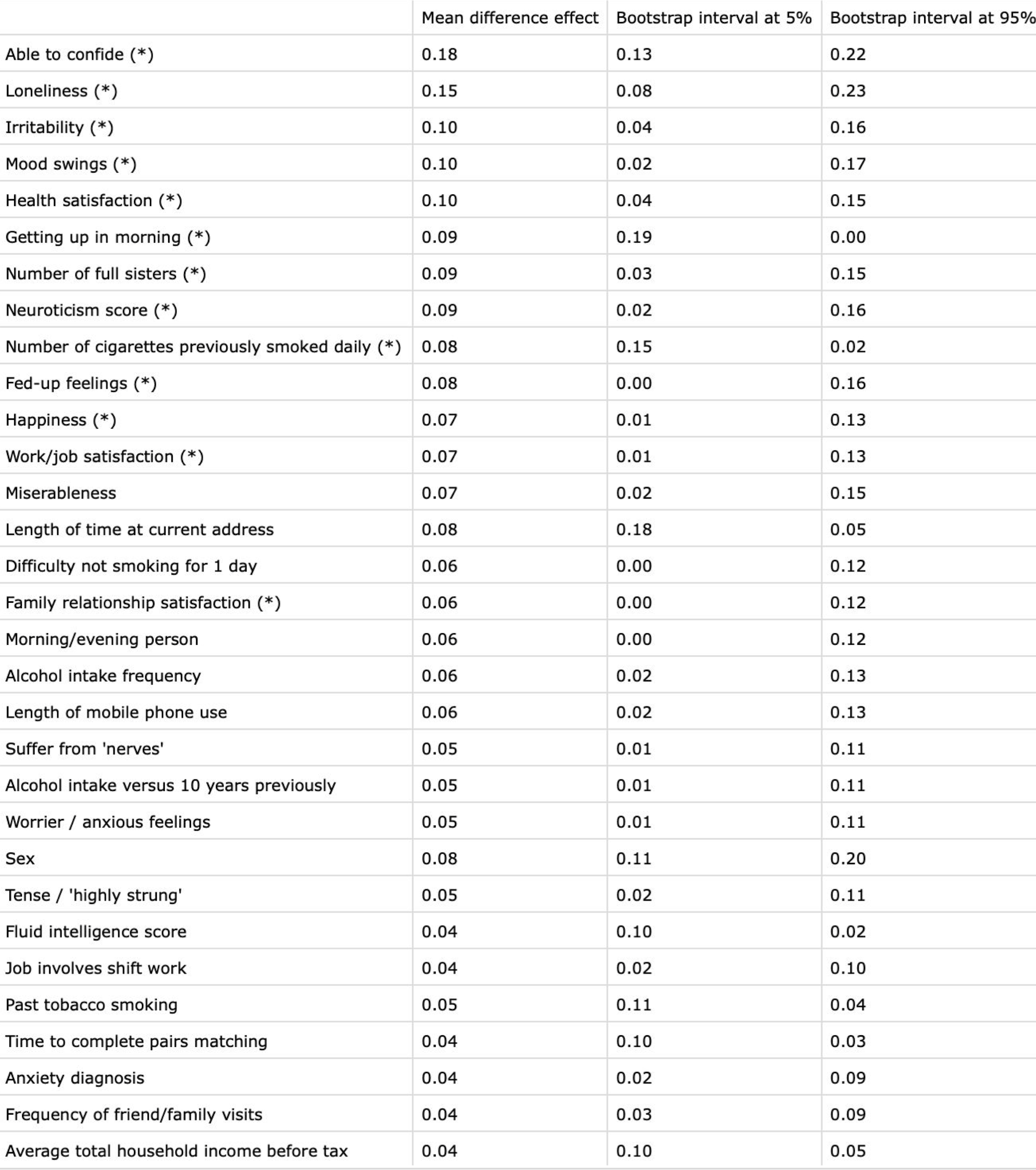


Table S3 (continued). Demographic profiling results for sMRI in detail.


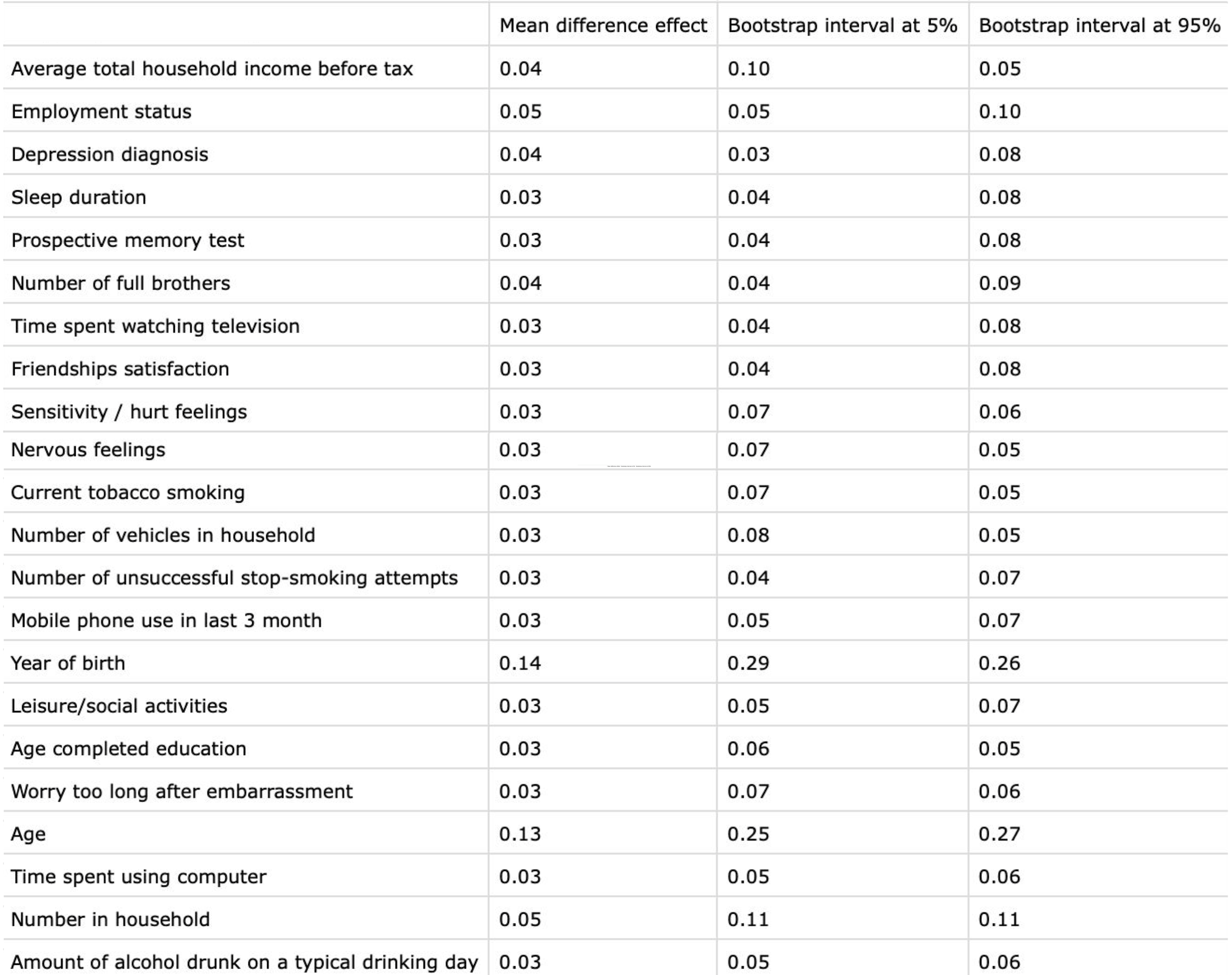

Supplement: SocialSupport_Supplementary_Materials_R1_bhab109 [file socialsupport_supplementary_materials_r1_bhab109.docx]
